# Genome-wide association mapping reveals a rich genetic architecture of stripe rust resistance loci in emmer wheat (*Triticum turgidum* ssp. *dicoccum*)

**DOI:** 10.1007/s00122-017-2957-6

**Published:** 2017-08-02

**Authors:** Weizhen Liu, Marco Maccaferri, Xianming Chen, Gaetano Laghetti, Domenico Pignone, Michael Pumphrey, Roberto Tuberosa

**Affiliations:** 1 Department of Crop and Soil Sciences, Washington State University, Pullman, WA 99164-6420 USA; 2 Department of Agricultural Sciences, University of Bologna, 40127 Bologna, Italy; 3 Wheat Health, Genetics, and Quality Research Unit, USDA-ARS, Pullman, WA 99164-6430 USA; 4 Department of Plant Pathology, Washington State University, Pullman, WA 99164-6430 USA; 5 CNR-Institute of Biosciences and Bioresources, 072006 Bari, Italy

## Abstract

*****Key message***:**

**SNP-based genome scanning in worldwide domesticated emmer germplasm showed high genetic diversity, rapid linkage disequilibrium decay and 51 loci for stripe rust resistance, a large proportion of which were novel.**

**Abstract:**

Cultivated emmer wheat (*Triticum turgidum* ssp. *dicoccum*), one of the oldest domesticated crops in the world, is a potentially rich reservoir of variation for improvement of resistance/tolerance to biotic and abiotic stresses in wheat. Resistance to stripe rust (*Puccinia striiformis* f. sp. *tritici*) in emmer wheat has been under-investigated. Here, we employed genome-wide association (GWAS) mapping with a mixed linear model to dissect effective stripe rust resistance loci in a worldwide collection of 176 cultivated emmer wheat accessions. Adult plants were tested in six environments and seedlings were evaluated with five races from the United States and one from Italy under greenhouse conditions. Five accessions were resistant across all experiments. The panel was genotyped with the wheat 90,000 Illumina iSelect single nucleotide polymorphism (SNP) array and 5106 polymorphic SNP markers with mapped positions were obtained. A high level of genetic diversity and fast linkage disequilibrium decay were observed. In total, we identified 14 loci associated with field resistance in multiple environments. Thirty-seven loci were significantly associated with all-stage (seedling) resistance and six of them were effective against multiple races. Of the 51 total loci, 29 were mapped distantly from previously reported stripe rust resistance genes or quantitative trait loci and represent newly discovered resistance loci. Our results suggest that GWAS is an effective method for characterizing genes in cultivated emmer wheat and confirm that emmer wheat is a rich source of stripe rust resistance loci that can be used for wheat improvement.

**Electronic supplementary material:**

The online version of this article (doi:10.1007/s00122-017-2957-6) contains supplementary material, which is available to authorized users.

## Introduction

Stripe rust (yellow rust) of wheat, caused by *Puccinia striiformis* f. sp. *tritici* (*Pst*), is a threat for wheat production in temperate areas throughout the world. Since the year 2000, stripe rust epidemics have been more common in the wheat growing areas previously believed to be too warm for widespread epidemic development as a consequence of the migration of temperature-adapted populations (Chen et al. 2010; Hovmøller et al. 2016). Populations of *Pst* with increased virulence and aggressiveness continue to spread and have defeated many major resistance genes and even combinations of these genes present in wheat varieties (Milus et al. 2009; Wan and Chen 2014). Although more than 70 officially designated yellow rust (*Yr*) genes and 100 temporarily named *Yr* genes or quantitative trait loci (QTL) (Rosewarne et al. 2013) have been identified in cultivated wheat and its wild relatives, susceptibility of widely grown cultivars is a global concern and nearly US$1 billion are lost to *Pst* annually (Beddow et al. 2015). Effective *Yr* resistance deployed in wheat varieties across most wheat growing regions of the world is needed to address the growing threat of stripe rust and minimize ongoing losses.

Cultivated emmer wheat (*Triticum turgidum* ssp. *dicoccum*), a self-pollinated allotetraploid species (2*n* = 4*x* = 28, AABB) with hulled grain, was one of the first crops domesticated in the world (Zohary and Hopf 2000). It was domesticated from wild emmer wheat (*Triticum turgidum* ssp. *dicoccoides*), which is also the progenitor of the two widely cultivated wheat species: tetraploid durum wheat (*Triticum turgidum* ssp. *durum*, 2*n* = 4*x* = 28, AABB) and hexaploid bread wheat (*Triticum aestivum* ssp. *aestivum*, 2*n* = 6*x* = 42, AABBDD) (Zeist et al. 1991; Luo et al. 2007; Faris et al. 2014). Previous studies have revealed that wild emmer wheat exhibits promising genetic diversity for resistance against *Pst* (Zaharieva et al. 2010) and direct hybridization between emmer wheat and cultivated wheat is highly fertile (Grama et al. 1984; Peng et al. 1999; Uauy et al. 2005), making it a promising genetic resource for enhancement of *Pst* resistance in cultivated wheat species. Several *Yr* resistance genes have been transferred from emmer wheat to cultivated wheat including *Yr15* (Gerechter-Amitai et al. 1989), *Yr30*/*Sr2* (McFadden 1930), *YrH52* (Peng et al. 1999), *Yr35*/*Lr52* (Dadkhodaie et al. 2011) and *Yr36* (Uauy et al. 2005; Fu et al. 2009). The emmer wheat-derived pleiotropic adult plant resistance (APR) gene *Sr2/Yr30* is arguably one of the most important resistance genes used in wheat breeding (McIntosh et al. 1995), especially given its importance in breeding for durable resistance to highly virulent ‘Ug99’ races of the stem rust pathogen (Singh et al. 2005). Despite the potential of emmer wheat to contribute novel alleles to wheat improvement, large collections of emmer wheat have been scarcely characterized using contemporary genomics resources and statistical genetics methods, which has hindered the exploitation of genetic variation in emmer wheat for cultivar improvement.

Most *Yr* genes and QTL were mapped by using bi-parental mapping populations that detect genetic variants between two, or at most several, parental accessions. Recently, linkage disequilibrium (LD)-based genome-wide association (GWAS) has attracted much interest, mostly in hexaploid bread wheat, as a method complementary to traditional bi-parental mapping to identify novel loci responsible for rust resistance in global germplasm collections. In wheat, GWAS investigation methods have been used for the first time to identify rust-resistance genome signals by Crossa et al. (2007) in hexaploid wheat and by Maccaferri et al. (2010) in tetraploid wheat. Since then, the method has been widely used to identify leaf rust (Kertho et al. 2015), stem rust (Yu et al. 2012; Letta et al. 2013; Laidò et al. 2015) and stripe rust resistance, to verify the effect of previously discovered *Yr* genes and QTL, and to assess the distribution of resistance loci in global germplasm (Tadesse et al. 2013; Zegeye et al. 2014; Maccaferri et al. 2015b; Naruoka et al. 2015; Bulli et al. 2016; Pasam et al. 2017).

A major advantage of GWAS approaches over bi-parental linkage mapping is that phenotype–genotype relationships in existing germplasm collections or natural populations may be explored for hundreds to thousands of accessions simultaneously, hence avoiding the resource intensive development of bi-parental genetic mapping populations. Association mapping enables researchers to use more genetic diversity and historical recombination events, which may result in higher mapping resolution than linkage mapping with a few hundred progeny (Zhu et al. 2008; Brachi et al. 2011) when high-density sequence-based markers are available (Wang et al. 2014b).

Association mapping has limitations, particularly as detection power depends heavily on the allele frequency distributions in a population. Rare alleles, with a frequency of ~5% or less, are not efficiently discovered by association mapping, unless their effects are large and can be validated through other information (Rafalski 2010). In addition, if samples in the population are not genetically homogeneous, population structure may cause spurious correlations between markers and traits resulting in elevated false-positive rates (Zhao et al. 2007). Recently, several statistical models have been developed to effectively control population structure as well as genetic relatedness among individuals in GWAS studies, including the mixed linear models (MLM) (Yu et al. 2005), compressed MLM (cMLM) (Zhang et al. 2010b), efficient mixed-model association (EMMA) (Kang et al. 2008), Settlement of MLM Under Progressively Exclusive Relationship (SUPER) model (Wang et al. 2014a) and fixed and random model circulating probability unification (FarmCPU) (Liu et al. 2016). LD decay rate, which relates to the mapping resolution of association analysis, is another important feature that determines the effectiveness of association mapping (Flint-Garcia et al. 2003). A faster LD decay rate in a population often implies a higher mapping resolution as long as a large number of genetic markers are available (Zhu et al. 2008). Compared with cultivated durum and bread wheat, emmer wheat populations may harbor more genetic variants and show a higher mapping resolution because populations have a longer history of recombination and have not been subject to the same extent of domestication bottlenecks (Salamini et al. 2002; Zaharieva et al. 2010). Sela et al. (2014) observed a rapid LD decay in a wild emmer wheat population of 128 accessions, which decayed to its background level within 1 cM. But until now, no study has targeted the whole-genome LD pattern in cultivated emmer wheat.

In this paper, we employed a panel of worldwide cultivated spring emmer wheat accessions, representing a large portion of the genetic diversity in the cultivated spring emmer wheat gene pool. Our objectives were to assess the genetic diversity, population structure and LD pattern of cultivated emmer wheat using high-density genome-wide SNP markers, identify stripe rust resistant/susceptible accessions, while exploring the genetic architecture of stripe rust resistance loci by GWAS experiments.

## Materials and methods

### Association mapping panel

A collection of 196 cultivated spring emmer wheat accessions, representing a large portion of the worldwide genetic diversity in the cultivated spring emmer wheat gene pool, was assembled from germplasm bank accessions originally collected and stored at the Institute of Biosciences and Bioresources, National Research Council of Italy (Volpe et al. 2005), Bari, Italy. The accessions were initially grown in observation rows at the University of Bologna experimental farm in Cadriano (44°33′N, 11°21′E, 32 m asl), Bologna, Italy, and then subjected to a 3-year single seed descent (SSD) purification step in 2009–2011. At the end of the third year of head-row advancement, the resulting selections (one to three for each original accession, depending on the phenotypic variability observed in the original accessions) where thus considered as genetically homogeneous and therefore amenable to subsequent genetic and phenotypic characterization. The selections were subjected to Illumina Infinium iSelect 90k wheat array genotyping and phenotyping for responses to *Pst* infection. After filtering for genotypic data quality and redundancy of genotypes, 176 accessions were further retained for population structure and kinship analyses (Supplemental File 1). Briefly, the genotypes can be grouped based on their geographic origins: group I: 22 accessions selected in Western European countries (Austria, Germany and United Kingdom); group II: 36 accessions derived from Southern Europe (Italy, Romania and Spain); group III: 28 accessions collected from Eastern European countries (Bulgaria, Georgia, Hungary, former Yugoslavia, Russia, Ukraine); group IV: 42 accessions cultivated in Eastern Africa (Ethiopia and Kenya); group V: one accession derived from North Africa (Morocco); group VI: five accessions selected in Southern Asia (India); group VII: two accessions bred in Eastern Asia (China); group VIII: 33 accessions collected from the Southern Levant and Western Asia (Afghanistan, Armenia, Iran, Syria and Yemen); and group IX: three accessions cultivated in Northern America (Canada and the USA) and group X: four accessions with unknown origins.

### Stripe rust evaluation at the adult plant stages in the field

All accessions were evaluated for *Pst* resistance in six field-based disease screening nurseries in Washington State (WA), US: Whitlow Farm (46°43′16.59″N 117°9′0.612″W, eastern WA) in 2014 (WHT14), Spillman Farm (46°43′47.1972″N 117°10′54.2568″W, eastern WA) in 2014 (SPM14) and 2015 (SPM15), Mount Vernon (48°25′16.3776″N 122°20′2.5692″W, western WA) in 2014 (MTV14) and 2015 (MTV15), and Central Ferry (46°38′9.816″N 117°47′24.0792″W, central WA) in 2015 (CLF15). Each environment was subject to moderate to severe *Pst* epidemic development each year with natural inoculum. The prevailing *Pst* races in each environment during 2014–2015 were described in Liu et al. (2017a, b): PSTv-37 predominated in all the environments apart from CLF15, and PSTv-52 was another important race detected in all environments except from MTV15.

At each location, approximately 150 seeds of each accession were planted in a 0.5-m row, spaced 0.3 m apart without replicates. The position of the 196 accessions was random in different field trails. To insure the uniformity of *Pst* inoculum across the trial, spring wheat cultivar ‘Avocet Susceptible’ (AvS) was planted every 20 rows as spreader rows, and another susceptible spring wheat variety, ‘Lemhi’, was planted around each plot as borders. Before planting, standard practices for fertilization and weed control common to the region were applied for field management. Two disease phenotypes were evaluated: infection type (IT) based on a 0–9 numerical scale and disease severity (SEV) rated as a percentage of disease area covering flag leaves (0–100%) (Line and Qayoum 1992). The stripe rust response in six environments was evaluated three times after the susceptible check ‘AvS’ had IT from 7 to 9 and SEV from 70 to 100%. The highest IT and SEV were used as the final phenotypes for each accession. Heading date (HD) and plant height (PH) traits for each accession were also recorded. Since the evaluation of *Pst* resistance may be influenced by flowering time, the accessions with very late HD were excluded when analyzing field nursery response to *Pst* races.

### Seedling resistance evaluation

Five *Pst* races (PSTv-14, PSTv-18, PSTv-37, PSTv-40 and PSTv-51) that are most common in US *Pst* populations, and one *Pst* race (PSTv-125) collected in Italy (Liu et al. 2017a), were used for seedling resistance screening of the emmer wheat panel under controlled conditions (Supplemental Table 1). PSTv-37 is widely distributed across the US (Wan and Chen 2014), PSTv-14 and PSTv-40 are common in the western US and PSTv-51 had the broadest spectrum of virulence. PSTv-18 is the least virulent race identified in the US so far, which is avirulent on all 18 *Yr* single-gene differentials. PSTv-125 was the most common race collected from both hexaploid and tetraploid wheat in Italy in 2014.

Seedling resistance to *Pst* was tested in controlled greenhouse conditions. Three seeds of each accession and susceptible check ‘AvS’ were planted in 96-well trays. Six *Pst* races (Supplemental Table 1), representing various virulence patterns, were chosen to test seedlings of this panel for all-stage resistance. Seedlings were grown under a diurnal temperature cycle of 20–24 °C and 15–18 °C at night with 16 h photoperiod for 10–14 days. At the two-leaf stage, plants were inoculated in a 4 °C dark dew chamber with 100% relative humidity for 24 h and then placed in a growth chamber. A core set of *Pst* differentials containing 18 *Yr* genes (Wan and Chen 2014) was included in each test to confirm the race identity. Infection type was recorded 16–20 days after inoculation. For each accession, three plants were recorded as one IT score if their reactions were the same. If reactions were different, we re-tested that accession. In addition, we re-tested the accessions with IT scores of 0–2 in order to validate their resistance reactions to the corresponding *Pst* races.

### Molecular profiling

Genomic DNA was extracted from a bulk of 25 one-week-old seedlings per accession using the DNeasy 96 Plant Kit (Qiagen GmbH, Hilden, Germany). The 196 accessions were genotyped using the Illumina^®^ iSelect 90K wheat SNP assay (Wang et al. 2014b) at the USDA-ARS Biosciences Research Laboratory, Fargo, ND. SNP marker genotype calling was performed using the GenomeStudio v2011.1 software package (Illumina, San Diego, CA, USA). The dataset filtering was carried out based on the following criteria: (1) markers showing residual heterozygosity were entered as missing values; (2) markers with less than 10% missing data and accessions with less than 20% missing data were retained; (3) markers with minor allele frequency (MAF) greater than 10% were retained; (4) one representative accession was retained for groups of accessions with genetic similarity equal to 1. A total of 176 lines and 5106 polymorphic SNPs with known map positions based on a high-density consensus map of tetraploid wheat (Maccaferri et al. 2015a) were used for population structure and kinship analyses. Molecular markers barc8 (Yaniv et al. 2015) and wMAS000005 (Distelfeld et al. 2006) that are widely used as markers for *Yr15* and *Yr30*/*Sr2,* and KASP_IWA6121 and KASP_IWA4096 (Naruoka et al. 2016) that flank *Yr5*, were profiled on this emmer wheat panel (Supplemental Table 2).

### Statistical analysis of phenotypic data

The broad sense heritability (*H* ^2^) of IT and SEV were calculated by a random effect model using PROC MIXED COVTEST in SAS v.9.3 (SAS Institute Inc., Cary, NC, USA) treating genotype, environment and genotype × environment interaction as random factors. The equation of *H* ^2^ is as follows (Piepho and Möhring 2007):

$$H^{2} = {{\sigma_{\text{G}}^{ 2} } \mathord{\left/ {\vphantom {{\sigma_{\text{G}}^{ 2} } {\left[ {\sigma_{\text{G}}^{2} + \sigma_{{{\text{E }} \times {\text{ G}}}}^{2} /n \, + \sigma_{\text{e}}^{ 2} /rn} \right]}}} \right. \kern-0pt} {\left[ {\sigma_{\text{G}}^{2} + \sigma_{{{\text{E }} \times {\text{ G}}}}^{2} /n \, + \sigma_{\text{e}}^{ 2} /rn} \right]}},$$

where *σ*_G_^2^ is the variance component of genotypes; *σ*_E × G_^2^ is the variance of the interaction of environment and genotype; *σ*_e_^2^ is the variance of residual; *n* is the number of environments and *r* is the number of replicates per environment.

Using the PROC ANOVA in SAS v.9.3, a one-way ANOVA and Tukey’s mean comparison tests were performed for IT and SEV among STRUCTURE-based subpopulations at a significance level of *P* < 0.05, where subpopulation was treated as fixed effect.

PROC CORR in SAS v.9.3 was used to compute the Pearson’s correlation coefficients. Correlations of IT and SEV among environments were computed to investigate the consistency of *Pst* responses across different environments.

Best linear unbiased predictors (BLUP) for each accession across environments was computed for traits including field-based *Pst* responses (IT and SEV), HD and PH, using PROC MIXED procedure in SAS v.9.3 (Wolfinger et al. 1997). In the PROC MIXED procedure, phenotypes of check ‘AvS’ were considered to be a fixed effect while environment and the interaction of environment and genotype were random effects.

### Genetic diversity and LD analyses

POWERMARKER v3.25 (Liu and Muse 2005) was used to calculate two genetic diversity parameters: genetic diversity and polymorphism information content (PIC). Genetic diversity is the probability of two randomly chosen alleles from the population being different (Weir and Cockerham 1996). PIC estimates the detection power and informativeness of the SNP markers (Botstein et al. 1980). The formula is ${\text{PIC}}_{j} = 1 - \sum_{i = 1}^{n} p_{i}^{2}$, where *p*_*i*_ is the frequency of the *i*th allele of the *j*th marker, *n* is the number of allele at the *j*th marker.

LD was measured as the squared correlation coefficient (*r* ^2^) between SNP pairs in the same chromosome using JMP^®^ Genomics 6.0 (SAS Institute Inc., Cary, NC, 2012). All 5106 mapped SNP markers were used in this LD analysis. To visualize the local LD patterns and overall LD decay rate, the intra-chromosomal pairwise *r* ^2^ estimates were plotted against inter-marker genetic distance (cM) (Fang et al. 2013; Rexroad and Vallejo 2009) and a non-parametric regression curve was fitted with smoothing spline, lambda = 10,000 function in JMP^®^ Genomics 6.0. The confidence interval of QTL was defined based on the intersection of the smoothing spline curve with the pairwise LD baseline (*r* ^2^ = 0.3) (Maccaferri et al. 2015b; Bulli et al. 2016).

### Association analyses

To minimize false-positive marker–trait associations (MTAs), population structure and genetic relatedness were factored into the GWAS analyses. Only SNP markers with known map position were loaded into HAPLOVIEW v4.2 (Barrett et al. 2005) to choose representative SNPs (haplotype tag-SNP). A total of 3361 tag-SNPs were chosen for calculating the marker-based kinship matrix (*K* matrix) based on the tagger function set at *r* ^2^ = 1.0, and 1422 tag-SNPs were further selected for calculating population structure (*Q* matrix) analysis using a setting of *r* ^2^ = 0.5. The population structure for this panel was determined by the Bayesian model-based clustering method in the software STRUCTURE v.2.3.4 (Pritchard et al. 2000; Falush et al. 2003). The parameter settings of STRUCTURE were: admixture model of population structure with correlated allele frequencies, 50,000 length of burn-in period, 100,000 number of Markov Chain Monte Carlo (MCMC) replications after burn-in, hypothetical subpopulations *k* setting from 1 to 10 with five iterations. STRUCTURE HARVESTER (Earl 2012) collated the output that was generated by STRUCTURE. An ad hoc statistic ∆*k*, on the basis of the rate of change in the logarithm of likelihood [ln *P*(*D*)] between successive *k* values, was used to detect the number of groups that best represented this population (Evanno et al. 2005). Kinship coefficients of all pairs of accessions were calculated based on the identity-by-state (IBS) genetic similarity (Kang et al. 2008).

Association mapping was conducted in the R package Genome Association and Prediction Integrated Tool (GAPIT) (Lipka et al. 2011) using all 5106 mapped polymorphic SNPs suitable for GWAS (MAF >0.10). Different association models were tested separately for the entire panel for seedling resistance and the subset panel for field resistance: (1) the general linear model (GLM) corrected for population structure using STRUCTURE membership (thereafter, *Q* GLM); (2) MLM (Yu et al. 2005) incorporating kinship as the random component (thereafter, *K* MLM); (3) MLM with *Q* matrix as the fixed factor and *K* matrix as the random factor (thereafter, *Q*+*K* MLM). Model selection was made based on the Bayesian information criterion values (BIC) computed by GAPIT (Supplemental Table 3). The *K* MLM model was chosen for the seedling response MTA analyses since it showed the best BIC values for the majority of *Pst* races (analysis based on all 176 accessions selected for GWAS). For the field-based adult plant responses, GWAS was conducted on a further subset of 109 accessions selected for narrow HD (14 day range). Thus *Q* and *K* matrices were re-calculated based on the genotypic data of 109 accessions. Model comparison was conducted and the *K* MLM model was chosen due to the best BIC values across the majority of environments (Supplemental Table 3). To control the impact of HD and PH on the level of stripe rust resistance in the field, HD and PH were included as covariates in the *K* MLM model for each of the six environments and the BLUP data across these six environments.

### Alignment of significant loci to previously published *Yr* genes and QTL

Liu et al. (2017a) characterized 93 loci associated with seedling and APR to *Pst* races in a worldwide collection of elite durum wheat. Straightforward map-based comparison of significant SNPs detected in this study with SNPs detected in elite durum wheat panel was performed by using a high-density SNP based on the tetraploid wheat consensus map (Maccaferri et al. 2015a).

Maccaferri et al. (2015b) constructed an integrated map including 225 previously reported *Yr* genes and QTL, SNPs, simple sequence repeat (SSR), diversity arrays technology (DArT) and express sequence tag (EST) markers. The map distances in the integrated map are based on the hexaploid wheat consensus map reported by Wang et al. (2014b), which is different from the tetraploid wheat consensus map (Maccaferri et al. 2015a) used in this study. The map positions of identified QTL in the tetraploid wheat consensus map and integrated map were reported in Supplemental Table 5, and the map positions of identified QTL in the integrated map were utilized to compare with the positions of previously published *Yr* genes in the integrated map, based on common shared markers. The use of alignment results is approximate and caution should be exercised because of the inherent limitations in the consensus map and integrated map.

## Results

### SNP marker quality, genomic distribution and genetic diversity

In total, 196 accessions of cultivated emmer wheat were genotyped using the 90K (81,587 actual markers) Illumina Infinium iSelect wheat SNP array. A total of 30,559 polymorphic SNPs with high-quality genotype calling were generated, of which 20,730 SNPs were mapped to the cultivated emmer wheat genome based on a high-density consensus map of tetraploid wheat (Maccaferri et al. 2015a). After filtering the markers with the criteria of MAF > 0.10 and missing data frequency <0.10, 5106 polymorphic SNP markers with known map positions were retained. Removing accessions with more than 20% missing marker frequency and genetic similarity equal to one, 176 genotypes were used for the following analyses. Marker distribution, MAF, number of alleles of each SNP marker, genetic diversity and PIC of each chromosome were calculated and presented in Supplemental Table 4.

In this population of cultivated emmer wheat, the B genome (3009 SNPs) had more polymorphic markers than the A genome (2097 SNPs), but differences in genetic diversity (B genome = 0.2976; A genome = 0.2802) and PIC (B genome = 0.2374; A genome = 0.2251) values were not statistically significant based on paired *t* tests. Polymorphic marker distributions ranged from 243 to 564 markers per chromosome, with an average density of 365 markers per chromosome. Chromosome 1B and 2B had the largest number of markers, and higher genetic diversity and PIC values than the genome-wide averages, while chromosome 3A had the smallest number of markers, and lower genetic diversity and PIC values. The overall genetic diversity and PIC values for this cultivated emmer wheat panel were 0.2895 and 0.2321, respectively.

To understand genetic diversity based on area of origin, the proportion of polymorphic loci, genetic diversity and PIC were calculated for each of the 10 origin groups (Table 1). Among the 10 groups, higher genetic diversity was observed in Western and Eastern European accessions than the others, with mean genetic diversity values of 0.2518 and 0.2497, and PIC values of 0.2044 and 0.2008, respectively. On the contrary, accessions in Eastern Asia showed the lowest genetic diversity and PIC values (0.1094 and 0.1082). The remaining regions including Southern Asia, Western Asia, Northern America, Eastern Africa and Southern Europe had intermediate levels of genetic diversity and PIC values.

**Table 1:** Summary of genetic diversity and polymorphism information content (PIC) values for a collection of 176 *Triticum turgidum* ssp. *dicoccum* accessions included in this study, originating from ten diverse regions of origin

Region	Country	Sample size	Polymorphic marker rate (%)	Mean value of genetic diversity	Mean PIC value
Eastern Africa	Ethiopia and Kenya	42	16.52	0.1937	0.1638
Northern Africa	Morocco	1	–	–	–
Eastern Asia	China	2	0.57	0.1094	0.1082
Southern Asia	India	5	7.77	0.1570	0.1263
Western Asia	Afghanistan, Armenia, Iran, Syria and Yemen	33	11.84	0.2143	0.1774
Eastern Europe	Bulgaria, Georgia, Hungary, former Jugoslavia, Russia and Ukraine	28	14.71	0.2497	0.2008
Southern Europe	Italy, Romania, and Spain	36	14.36	0.2004	0.1653
Western Europe	Austria, Germany, and United Kingdom	22	17.70	0.2518	0.2044
Unknown	Unknown country	4	17.54	0.2419	0.1909
Northern America	Canada, the United States	3	12.93	0.1600	0.1282
Total/grand mean		176	12.66	0.2895	0.2321

### Population structure, kinship and LD

Population structure was analyzed by Bayesian-based structure analysis using 1422 non-redundant markers (LD *r* ^2^ = 0.5) across the whole genome. A clear peak at *k* = 3 revealed that the optimal population structure for further analyses was to divide this panel into three main subgroups. Subpopulation one, two and three contained 45, 78 and 53 accessions, respectively. The majority of accessions (71%) collected from Africa (Ethiopia, Kenya and Morocco) were grouped into subpopulation one (“African-subpopulation”), while 92% of accessions from Southern Europe and 77% of accessions from Western Europe were grouped into subpopulation two (“European-subpopulation”). All accessions from Eastern Asia and 82% of accessions from Western Asia contributed to subpopulation three (“Asian-subpopulation”).

**Fig. 1:**
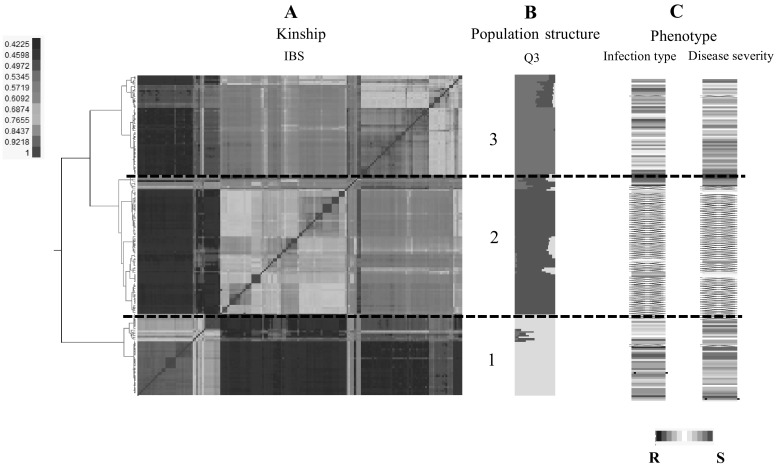
Population structure analysis of 176 cultivated emmer wheat accessions. **a** Heat map of kinship matrix on the basis of identical-by-state (IBS). **b** Population structure summary plot (*k* = 3) of membership coefficients using STRUCTURE v.2.3.4. From the *bottom* to the *top*, they are subpopulation 1, 2 and 3. The *horizontal dashed lines* separated the 176 accessions into three subpopulations according to structure membership coefficients. **c** Cell plot displayed phenotypic reactions to *Puccinia striiformis* f. sp. *tritici* (*Pst*) at the adult stages in STRUCTURE-based subpopulations. *Red* to *white* to *blue lines* indicate reactions to *Pst* changed from susceptibility to intermediate to resistance to stripe rust. *Black lines* indicate the accessions that displayed winter habit or had very late heading date that excluded from the GWAS analysis at the adult stages. The order of individuals in **a**–**c** were arranged according to their IBS-based genotypic distance

Genetic relatedness was also calculated using the IBS coefficient among all pairs of individuals. The IBS allele-sharing matrix (“kinship matrix”) clearly mirrored subpopulation ancestry as distinguished by the population structure analysis (Fig. 1). Only two accessions in STRUCTURE-based subpopulation two were clustered into IBS-based subpopulation three. Accessions in each subpopulation were highly related, and the mean IBS coefficients within the three subpopulations were 0.86, 0.70 and 0.82, for subpopulations one, two and three, respectively.

**Fig. 2:**
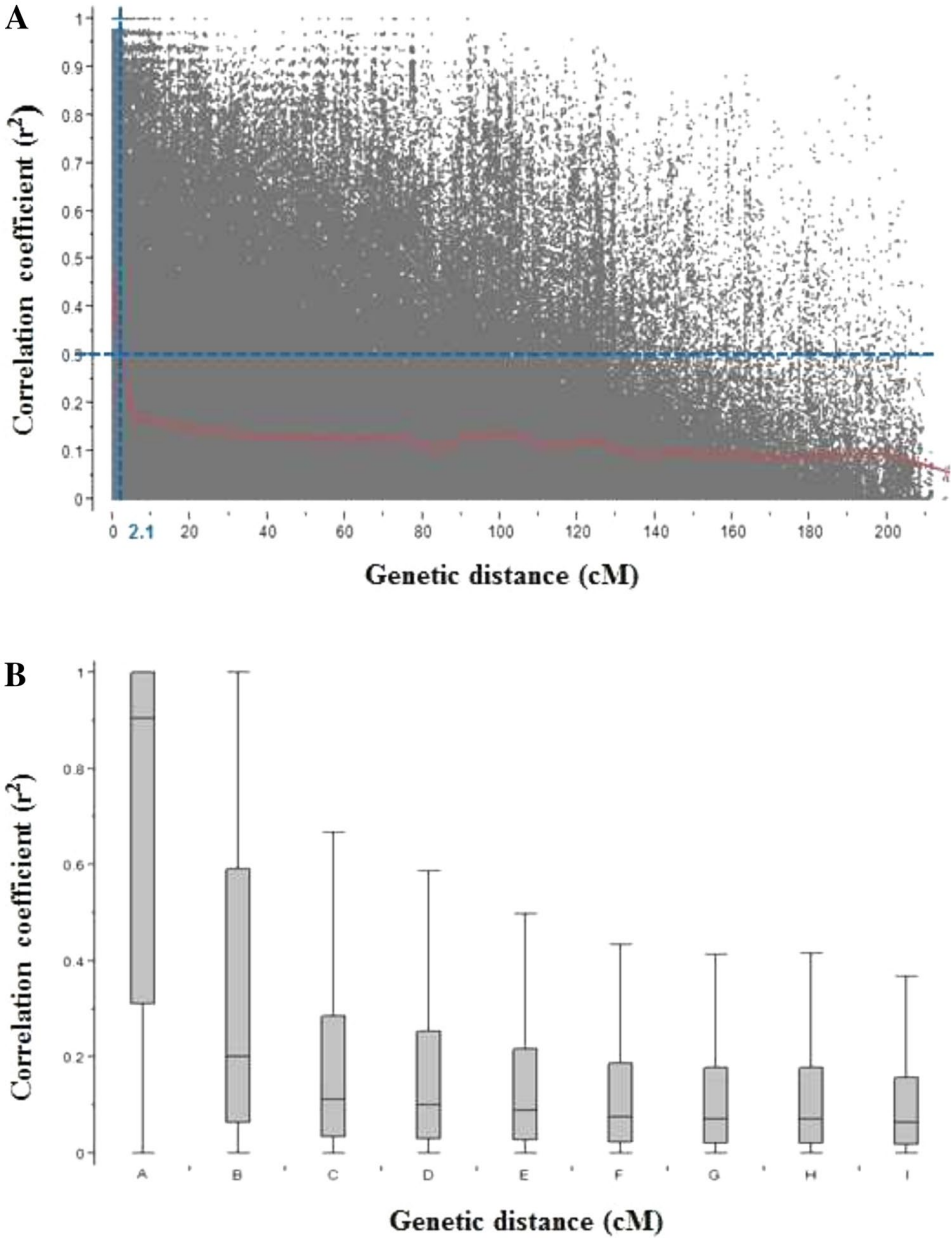
Genome-wide average linkage disequilibrium (LD) decay plot for 176 cultivated emmer wheat accessions based on 5106 single nucleotide polymorphism (SNP). **a** Plot of pairwise SNP LD *r* ^2^ value as a function of inter-marker genetic distances (cM). **b** Box-plot of LD *r* ^2^ between pairs of SNPs against incremental classes of genetic distances (cM). *A*, *B*, *C*, *D*, *E*, *F*, *G*, *H* and *I* mean inter-marker genetic distances at 0, 0.1–1, 1–5, 5–10, 10–20, 20–30, 30–40, 40–50 and >50 cM

The mean genome-wide LD pattern of the 176 cultivated emmer wheat genotypes is graphically displayed by scatter and box plots of pairwise LD *r* ^2^ over genetic distance in cM (Fig. 2). The box-plot of LD *r* ^2^ distribution (Fig. 2b) showed that the median LD *r* ^2^ of the completely linked marker pairs was 0.91 with interquartile ranging from 1.00 to 0.31. In the next marker group (tightly linked markers separated <1.0 cM), the median LD *r* ^2^ dramatically decreased to 0.20, indicating an average of 78.02% LD decay rate within a 1.0 cM genetic distance. For pairs of markers with genetic distances of 1.0–5.0 cM, the median LD *r* ^2^ reduced to 0.11. Linkage disequilibrium *r* ^2^ started to decrease slowly after pairwise marker genetic distances greater than 5.0 cM. The average LD *r* ^2^ dropped from 0.1 to 0.06 when genetic distances of pairwise markers increased from 5.0 to >50.0 cM. Similar LD decay trends were determined when fitting the smoothing spline curve into the scatter-plot of LD *r* ^2^ distribution (Fig. 2a). The baseline (*r* ^2^ = 0.3) intersection with the smoothing spline curve was at 2.1 cM, which was used to determine the QTL coverage regions with marker distance confidence intervals of ±2.1 cM from the peak of the significant associations.

### Seedling response to six *Pst* races

**Fig. 3:**
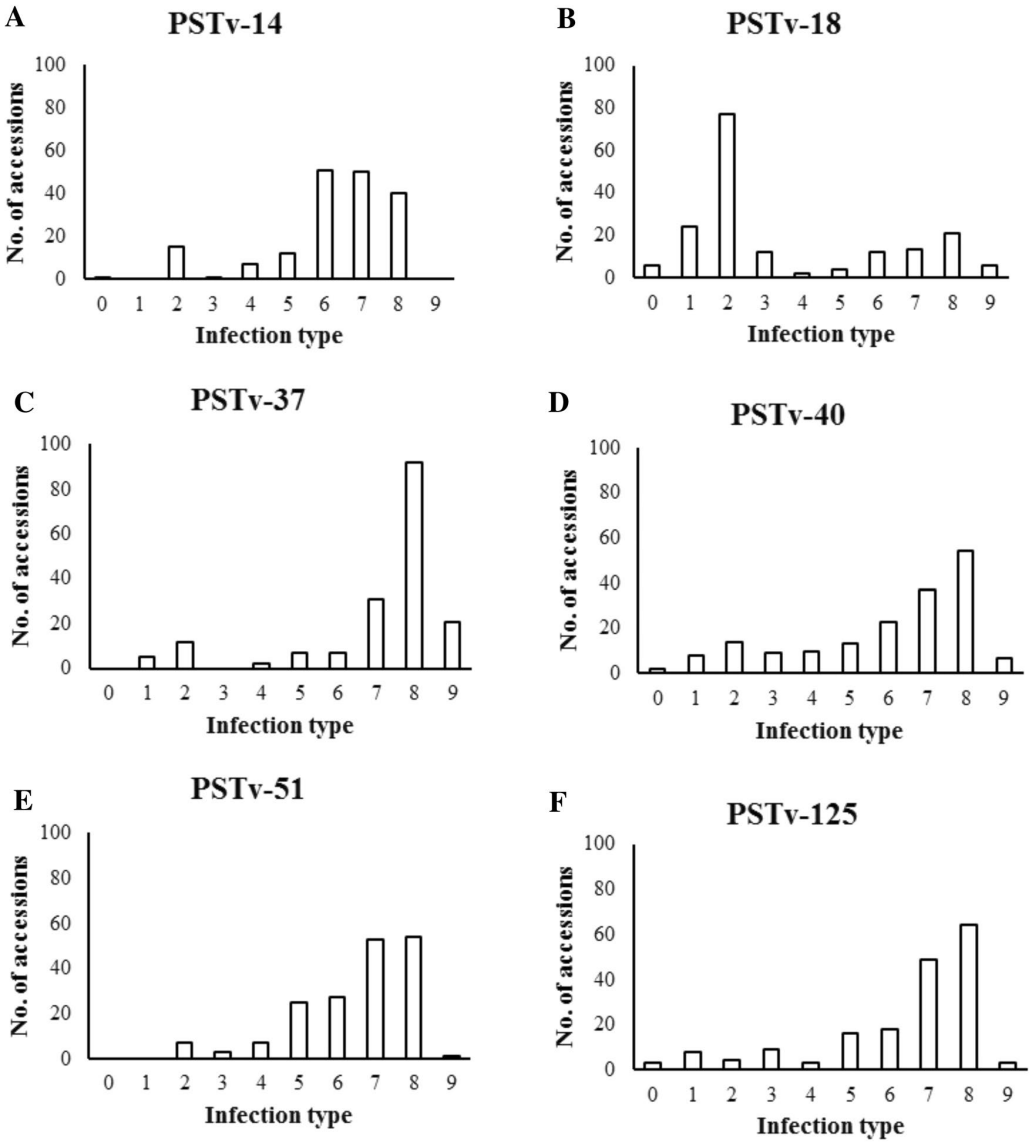
Distributions of infection type of 176 cultivated emmer wheat accessions at the seedling stage for **a** PSTv-14, **b** PSTv-18, **c** PSTv-37, **d** PSTv-40, **e** PSTv-51 and **f** PSTv-125

Seedling responses showed highly significant differences (*P* < 0.0001) among accessions within each race. The IT distributions of all *Pst* races summarized in Fig. 3 were skewed toward susceptible scores (IT = 7–9), except for the most avirulent race PSTv-18. For races PSTv-14, PSTv-37, PSTv-40, PSTv-51 and PSTv-125, susceptible reactions (IT = 7–9) were observed in 51, 81, 55, 61 and 66% accessions of this emmer wheat panel, respectively. A higher proportion (60%) of accessions exhibited highly resistant reactions (IT = 0–2) to PSTv-18 than the other races, while intermediate (IT = 3–6) and susceptible reactions to PSTv-18 were 17 and 23%, respectively. Five accessions in subpopulation one were resistant to all six *Pst* races, and 14 accessions in subpopulation two were susceptible to all races (Supplemental File 1).

The effect of population structure was significant for responses to *Pst* races. Accessions in subpopulation one (African germplasm) are more resistant than those in subpopulation two (European germplasm) and three (Asian germplasm) in responses to all *Pst* races tested in this study except for PSTv-18 (Table 2). The mean IT of accessions in subpopulation two and three are significantly (*P* < 0.05) higher than that of accessions in subpopulation one. The accessions in subpopulation two were more susceptible to PSTv-18 than accessions in subpopulation one and three. The mean IT of subpopulation two was significantly (*P* < 0.05) different from subpopulation one and three, but the mean IT of subpopulations one and three did not show a significant difference.

**Table 2:** Phenotypic reactions to *Puccinia striiformis* f. sp. *tritici* (*Pst*) of the 176 *Triticum turgidum* ssp. *dicoccum* accessions included in this study, grouped based on STRUCTURE (corresponding to subpopulations of genetically related individuals)

	STRUCTURE-base subpopulation		
	One (African origin)	Two (European origin)	Three (Asian origin)
*Pst* race (seedling stage)			
PSTv-14	4.71^a^	6.95^b^	6.34^b^
PSTv-18	2.84^a^	4.97^b^	2.04^a^
PSTv-37	5.04^a^	8.00^b^	7.49^b^
PSTv-40	4.20^a^	7.21^b^	5.74^c^
PSTv-51	5.36^a^	7.01^b^	6.68^b^
PSTv-125	4.64^a^	7.09^b^	6.75^b^
BLUP (adult stage)			
Infection type	4.79	4.84	4.27
Disease severity	42.04^a^	31.85^ab^	28.51^b^

The phenotypic data were averaged across individual subpopulations. The letter of superscript showed significant differences of mean infection type and disease severity between individual subpopulations at *P* value <0.05

### Field response to *Pst* in six field environments

Accessions in this cultivated emmer wheat panel showed substantial diversity in morphological and physiological traits under field conditions, including substantial variations in HD (a 32-day range). Eleven accessions in subpopulation two displayed winter habit in our field screening nurseries, and 55 accessions (53 accessions in subpopulation two) had very late HD, associated to IT and SEV = 0. In order to reduce the influence of late HD on the analysis and interpretation of field stripe rust response evaluation, 109 accessions were selected from the original collection of 176 accessions based on HD consistently comprised within a 14-day range.

**Fig. 4:**
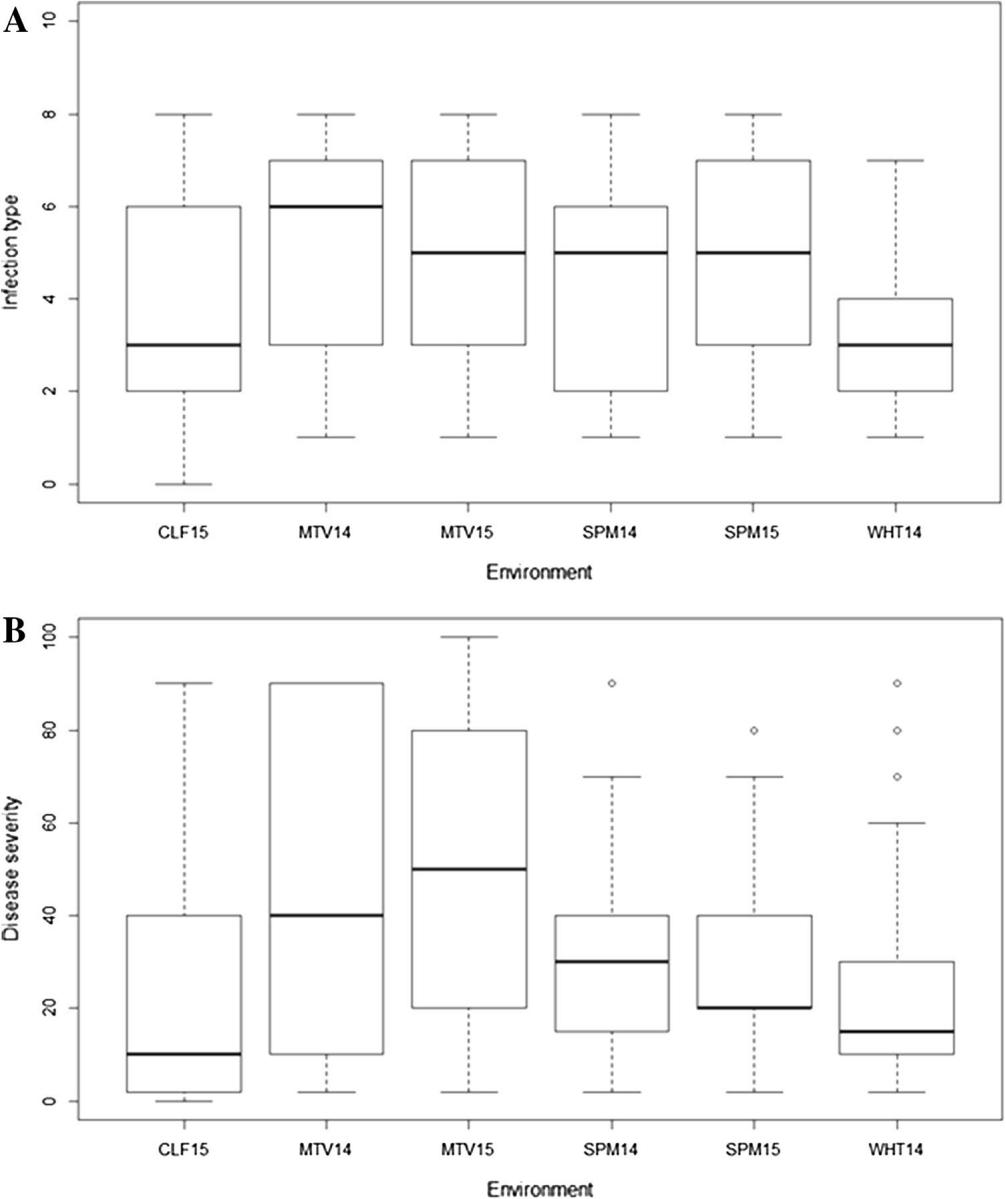
Distributions of field response to *Puccinia striiformis* f. sp. *tritici* of 109 cultivated emmer wheat accessions evaluated at adult stage in six environments in Washington state, US. **a** Infection type and **b** disease severity

The IT and SEV of 109 accessions at the adult stage were presented in Supplemental File 1. The distributions of IT and SEV of the six environments were all skewed toward resistance, and the cultivated emmer wheat population exhibited a higher level of resistance in CLF15 and WHT14 compared to the other four environments (Fig. 4). Based on the BLUP values of IT across six environments, about 25% of accessions (28 in total) were highly resistant (mean SEV = 14%), 69% (75 accessions) were intermediate (mean SEV = 39%) and 6% (6 accessions) were highly susceptible (mean SEV = 64%).

The heritability values for IT and SEV, calculated across the six environments, were 0.921 and 0.919. The high heritability indicated limited environmental variation compared to genotypic variation across the six environments. Pearson’s correlation coefficients of IT and SEV (Table 3) recorded in the six environments were all highly significant (*P* < 0.0001) with the range from 0.60 to 0.77 for IT and 0.57 to 0.82 for SEV. The correlation coefficients between IT and SEV within the same environment were moderate to high, ranging from 0.50 to 0.91. The five accessions that were resistant to all six *Pst* races at the seedling stages were also resistant based on their evaluation over six field environments at the adult stages.

**Table 3:** Pearson’s correlation coefficients of response to stripe rust of 109 cultivated spring emmer wheat accessions as evaluated in six environments (field trials) in Washington state

	SPM15	MTV15	CLF15	SPM14	WHT14	MTV14
IT vs. IT^a^						
SPM15		0.74	0.64	0.75	0.67	0.67
MTV15			0.68	0.63	0.65	0.77
CLF15				0.70	0.70	0.60
SPM14					0.74	0.67
WHT14						0.72
MTV14						
SEV vs. SEV^b^						
SPM15		0.70	0.57	0.66	0.70	0.68
MTV15			0.69	0.64	0.76	0.82
CLF15				0.72	0.66	0.66
SPM14					0.72	0.71
WHT14						0.79
MTV14						
IT vs. SEV^c^						
SPM15	**0.50** ^d^	0.58	0.50	0.62	0.44	0.61
MTV15	0.58	**0.89**	0.61	0.62	0.66	0.78
CLF15	0.52	0.66	**0.84**	0.71	0.57	0.63
SPM14	0.48	0.52	0.57	**0.78**	0.55	0.63
WHT14	0.53	0.65	0.67	0.70	**0.70**	0.74
MTV14	0.62	0.75	0.56	0.64	0.69	**0.91**

^a^ Comparisons between the infection type of different environments. SPM15 = Spillman Farm 2015; MTV15 = Mount Vernon 2015; CLF15 = Central Ferry 2015; SPM14 = Spillman Farm 2014; WHT14 = Whitlow Farm 2014; MTV14 = Mount Vernon 2014

^b^ Comparisons between the disease severity of different environments

^c^ Comparison between the infection type and disease severity of different environments

^d^ Correlation coefficients between IT and SEV of the same environment were labeled in bold and underlined

The *P* values of all the Pearson’s correlation coefficients in the table are smaller than 0.0001 (*P* < 0.0001)

### Association analyses for race-specific resistance

A mixed linear model with *K* as a covariate was determined to be the optimal model based on the BIC values. The ITs of 176 accessions in response to races PSTv-14, PSTv-18, PSTv-37, PSTv-40, PSTv-51 and PSTv-125 were used to identify race-specific resistance MTAs. A total of 57 SNP markers within 37 distinct loci on all 14 chromosomes were identified to be significantly associated (*P* < 0.001) with seedling resistance. The phenotypic variance (*R* ^2^) explained by each of these MTAs ranged from 3.2 to 7.5% (Table 4).

**Table 4:** Putative resistance genes that were significantly (*P* < 0.001) associated with responses to races PSTv-14, PSTv-18, PSTv-37, PSTv-40, PSTv-51 and PSTv-125 of *Puccinia striiformis* f. sp. *tritici* (*Pst*) in seedling tests under controlled greenhouse conditions

*Yr* gene^a^	Chrom^b^	Confidence interval (cM)^c^	Tag-SNP^d^	Allele^e^	Associated SNP^f^	*R* ^2^ (%)^g^	To *Pst* race^h^
*YrTtd*-*1AS*	1AS	17.7–21.9	IWB22778	**C**/T	–	4.3	PSTv-37
*YrTtd*-*1AL*	1AL	86.2–90.4	IWB27332	**C**/T	–	5.2	PSTv-37
*YrTtd*-*1BS*	1BS	35.0–39.2	IWB47025	C/**T**	IWA7219, IWB14377, IWB47026, IWB49800, IWB62417, IWB64056	4.5–4.8	PSTv-40
*YrTtd*-*1BL*	1BL	81.8–86.0	IWB35698	A/**G**	–	4.6	PSTv-37
*YrTtd*-*2AL*	2AL	160.1–164.3	IWB67229	**C**/T	–	5.8	PSTv-37
*YrTtd*-*2BS*	2BS	25.6–29.8	IWB7081	C/**T**	–	5.7	PSTv-125
***YrTtd*** **-** ***2BL.1***	2BL	138.3–142.5	IWB48012	A/**G**	–	5.5–6.7	PSTv-37, PSTv-40, PSTv-51
*YrTtd*-*2BL.2*	2BL	163.8–168.0	IWB59983	A/**G**	–	6.1	PSTv-125
*YrTtd*-*3AL*	3AL	108.4–112.6	IWB71901	C/**T**	IWB70903, IWB70904	5.5–7.5	PSTv-51
*YrTtd*-*3BS.1*	3BS	84.3–88.5	IWA7905	C/**T**	IWB22863, IWB4227, IWB63776, IWB65606	4.5–4.9	PSTv-40
*YrTtd*-*3BL.1*	3BL	95.1–99.3	IWB37522	**C**/T	IWB39508, IWA6510	5.1–6.2	PSTv-37
*YrTtd*-*3BL.2*	3BL	147.8–152.0	IWB59536	C/**T**	–	6.0	PSTv-51
*YrTtd*-*3BL.3*	3BL	185.5–189.7	IWB10521	**A**/G	–	4.4	PSTv-40
*YrTtd*-*4AS*	4AS	23.1–27.3	IWB55738	A/**G**	–	5.3	PSTv-40
***YrTtd*** **-** ***4AL.1***	4AL	160.7–164.9	IWA1034	C/**T**	–	4.7–6.9	PSTv-40, PSTv-51
*YrTtd*-*4BS*	4BS	30.8–35.0	IWB56078	C/**T**	–	3.4	PSTv-18
***YrTtd*** **-** 4BL.1*	4BL	69.9–74.1	IWA1641	C/**T**	–	4.4–6.6	PSTv-37, PSTv-125
***YrTtd*** **-** 4BL.2*	4BL	116.7–120.9	IWB67499	**A**/G	–	5.1–6.3	PSTv-14, PSTv-37, PSTv-125
*YrTtd*-*5A*	5A	61.7–65.9	IWB46475	A/**G**	–	5.8	PSTv-37
*YrTtd*-*5AL.1*	5AL	140.4–144.6	IWA1829	A/**G**	–	7.8	PSTv-51
*YrTtd*-*5AL.2*	5AL	181.1–185.3	IWB67141	A/**G**	–	3.9	PSTv-37
*YrTtd*-*5BS.1*	5BS	36.1–40.3	IWB10728	C/**T**	IWB9675	4.6–7.0	PSTv-40
*YrTtd*-*5BS.2*	5BS	41.9–46.1	IWB66991	**C**/T	–	4.3	PSTv-40
*YrTtd*-*5BL.1*	5BL	72.7–76.9	IWB40681	A/**G**	–	4.3	PSTv-40
*YrTtd*-*5BL.2*	5BL	122.8–127.0	IWA7733	A/**C**	–	3.2	PSTv-18

*Yr* gene^a^	Chrom^b^	Confidence interval (cM)^c^	Tag-SNP^d^	Allele^e^	Associated SNP^f^	*R* ^2^ (%)^g^	Associated *Pst* race^h^
***YrTtd*** **-** ***6AS.1***	6AS	9.2–13.4	IWB63861	**C**/T	–	4.3–4.9	PSTv-18, PSTv-37, PSTv-40
*YrTtd*-*6AS.2*	6AS	19.3–23.5	IWB63758	A/**C**	–	4.5	PSTv-40
*YrTtd*-*6AL.1*	6AL	91.2–95.4	IWB9468	**A**/G	–	6.1	PSTv-37
*YrTtd*-*6AL.2*	6AL	114.1–118.3	IWB72189	**A**/G	–	4.0	PSTv-37
*YrTtd*-*6BS*	6BS	69.4–73.6	IWB60487	**C**/T	IWB47211	4.6–4.7	PSTv-40
*YrTtd*-*6BL*	6BL	74.4–78.6	IWB12289	C/**T**	–	4.8	PSTv-14
***YrTtd*** **-** 7AS*	7AS	6.6–10.8	IWB61392	C/**T**	–	5.1–7.2	PSTv-14, PSTv-37, PSTv-125
*YrTtd*-*7AL.1*	7AL	117.6–121.8	IWB21459	A/**G**	–	4.6	PSTv-40
*YrTtd*-7AL.2*	7AL	132.2–136.4	IWA1944	**A**/C	–	4.9	PSTv-14
*YrTtd*-*7BS.1*	7BS	28.1–32.3	IWB71730	C/**T**	–	3.9	PSTv-37
*YrTtd*-*7BS.2*	7BS	60.6–64.8	IWB13775	A/**G**	IWA3129, IWB41451, IWB70254, IWB69577	5.7–6.2	PSTv-37
*YrTtd*-*7BL*	7BL	91.9–96.1	IWB69807	A/**C**	–	4.5	PSTv-37

^a^ Putative *Yr* loci that have significant association with seedling response to multiple *Pst* races are given in bold. *Yr* loci that have significant association with seedling and field responses are marked by asterisks

^b^ Chromosome positions of putative *Yr* loci are based on the tetraploid wheat consensus map (Maccaferri et al. 2015a)

^c^ Confidence intervals are determined by confidence intervals of ± 2.1 cM from the peak of the significant associations

^d^ Tag-SNPs are the most significant SNP in the confidence interval of putative genes

^e^ Resistance-associated alleles of tag-SNPs are highlighted in bold

^f^ Significant SNPs associated to the same *Pst* races as the tag-SNP and falling into the confidence intervals of putative genes

^g^ Phenotypic variations explained by the tag-SNPs

^h^ *Pst* races that SNP markers are significantly (*P* < 0.001) associated with

MTAs can be categorized into two groups. The first group consisted of loci associated with resistance to multiple races. Six loci mapped on chromosomes 2BL, 4AL, 4BL, 6AS and 7AS were included in this group: *YrTtd*-*2BL.1*, *YrTtd*-*4AL.1*, *YrTtd*-*4BL.1*, *YrTtd*-*4BL.2*, *YrTtd*-*6AS.1* and *YrTtd*-*7AS*. Since this group of MTAs was consistently effective against multiple races, they might be more broadly useful in future germplasm enhancement and breeding efforts.

The second group included resistance loci significantly associated with only one *Pst* race. *YrTtd*-*6BL* and *YrTtd*-*7AL.2* showed significant association with US race PSTv-14. *YrTtd*-*1AS*, *YrTtd*-*1AL*, *YrTtd*-*1BL*, *YrTtd*-*2AL*, *YrTtd*-*3BL.1*, *YrTtd*-*5A*, *YrTtd*-*5AL.2*, *YrTtd*-*6AL.1*, *YrTtd*-*6AL.2*, *YrTtd*-*7BS.1*, *YrTtd*-*7BS.2* and *YrTtd*-*7BL* were significantly associated with resistance to US race PSTv-37. Ten loci were detected to be associated with resistance to US race PSTv-40, including *YrTtd*-*1BS*, *YrTtd*-*3BS.1*, *YrTtd*-*3BL.3*, *YrTtd*-*4AS*, *YrTtd*-*5BS.1*, *YrTtd*-*5BS.2*, *YrTtd*-*5BL.1*, *YrTtd*-*6AS.2*, *YrTtd*-*6BS* and *YrTtd*-*7AL.1*. Three loci, *YrTtd*-*3AL*, *YrTtd*-*3BL.2* and *YrTtd*-*5AL.1*, showed significant association with resistance to US race PSTv-51. PSTv-18, the least virulent *Pst* race, was used to identify the *Yr* resistance loci that were effective against relatively avirulent *Pst* races but have been defeated by more virulent *Pst* races. Two loci, *YrTtd*-*4BS* and *YrTtd*-*5BL.2*, were detected in this population conferring resistance to PSTv-18. Two loci, *YrTtd*-*2BS* and *YrTtd*-*2BL.2*, were exclusively associated with resistance to the virulent race of Italian origin, PSTv-125.

### Association analyses for resistance in field environments

To identify MTAs across field environments effective against contemporary US *Pst* populations at the adult plant stage, association tests were carried out separately for each of six field environments and also using BLUPs generated from data combined across the six environments. Significant (*P* < 0.05) Pearson’s correlations were observed between IT and HD (*r* = 0.24), IT and PH (*r* = 0.21), SEV and HD (*r* = 0.45), and SEV and PH (*r* = 0.41). Therefore, HD and PH were incorporated into MLM + *K* model as two covariates.

In total, 18 loci represented by 24 SNPs were significantly associated (*P* < 0.001) with IT and/or SEV in the field nurseries. The phenotypic variance explained by individual loci ranged from 7.2 to 12.5%. Without considering the interaction effects, the cumulative phenotypic variation explained by all 18 loci was 56.7% for BLUP_IT and 66.1% for BLUP_SEV. Four of these loci were also significantly associated with resistance in the seedling tests. These all-stage resistance loci were *YrTtd*-*4BL.1*, *YrTtd*-*4BL.2*, *YrTtd*-*7AS* and *YrTtd*-*7AL.2* highlighted in Table 4. The cumulative phenotypic variance explained by these loci was 10.1% for BLUP_IT and 11.2% for BLUP_SEV. Among these seedling resistance loci effective under field conditions, *YrTtd*-*4BL.1*, *YrTtd*-*4BL.2* and *YrTtd*-*7AS* were significantly associated with resistance in multiple field environments. The remaining 14 loci on chromosomes 1A, 1B, 2AS, 2BS, 3BS, 6BS, 7AL and 7BL were significantly associated with adult plant responses in the field, but not seedling responses, and may represent APR loci (Table 5). *QYrTtd*-*7AL.1* tagged by IWB25121 and *QYrTtd*-*7AL.2* tagged by IWA501 are mapped to chromosome 7AL with a genetic distance of 3.6 cM. Haplotype analysis showed these two QTL are not in LD (*r* ^2^ = 0.11), and *QYrTtd*-*7AL.1* is associated with SEV while *QYrTtd*-*7AL.2* is associated with IT, indicating that they are likely distinct QTL.

**Table 5:** Putative quantitative trait loci (QTL) significantly (*P* < 0.001) associated with response to stripe rust in field environments

QTL	Chrom^a^	Confidence interval (cM)^b^	Tag-SNP^c^	Allele^d^	Associated SNP^e^	*R* ^2^ (%)^f^	Field environment^g^
*QYrTtd*-*1A*	1A	50.9–55.1	IWA1279	**C**/T	IWB49698	8.5	CLF15_SEV
*QYrTtd*-*1BS*	1BS	4.0–8.2	IWB50501	A/**G**	–	7.4	WHT14_SEV
*QYrTtd*-*1BL*	1BL	103.9–108.1	IWB69464	C/**T**	–	9.5	MTV15_IT
*QYrTtd*-*2AS*	2AS	61.3–65.5	IWB1046	A/**C**	–	9.0	SPM15_IT
*QYrTtd*-*2AL*	2AL	105.6–109.8	IWB4635	**C**/T	–	8.1–11.2	MTV15_IT, MTV15_SEV
*QYrTtd*-*2BS.1*	2BS	7.9–12.1	IWB40673	C/**T**	–	8.1–9.9	CLF15_SEV
*QYrTtd*-*2BS.2*	2BS	74.7–78.9	IWB39220	A/**G**	–	8.2	MTV14_SEV
*QYrTtd*-*3BS.1*	3BS	46.8–51.0	IWB63252	**C**/T	–	9.0–9.1	MTV15_IT, SPM15_IT
*QYrTtd*-*3BS.2*	3BS	75.0–79.2	IWB124	A/**G**	IWA5813, IWB25636, IWB32812, IWB50708	10.9	MTV14_SEV
*QYrTtd*-*6BS.1*	6BS	5.9–10.1	IWB23395	C/**T**	–	8.4–9.4	MTV15_IT, BLUP_IT
*QYrTtd*-*6BS.2*	6BS	16.2–20.4	IWB27151	A/**G**	–	7.5	WHT14_SEV
*QYrTtd*-*7AL.1*	7AL	190.9–195.1	IWB25121	A/**G**	–	10.5	WHT14_IT
*QYrTtd*-*7AL.2*	7AL	198.7–202.9	IWA501	C/**T**	–	9.3	SPM15_IT
*QYrTtd*-*7BL*	7BL	191.1–195.3	IWB72249	**A**/G	–	10.1	MTV15_IT

^a^ Chromosome positions of the identified QTL are based on the tetraploid wheat consensus map (Maccaferri et al. 2015a)

^b^ Confidence intervals are determined by confidence intervals of ±2.1 cM from the peak of the significant associations

^c^ Tag-SNPs are the most significant SNP in the confidence interval of the identified QTL

^d^ Resistance-associated alleles of tag-SNPs are given in bold

^e^ Significant SNPs associated with the same *Pst* races as the tag-SNP and fall into the confidence intervals of putative genes

^f^ Phenotypic variations explained by the tag-SNPs

^g^ Environments where the marker–trait association are discovered. SPM15 = Spillman Farm 2015; MTV15 = Mount Vernon 2015; CLF15 = Central Ferry 2015; SPM14 = Spillman Farm 2014; WHT14 = Whitlow Farm 2014; MTV14 = Mount Vernon 2014

### Distributions of *Pst* resistance alleles in subpopulations and their influences on *Pst* reaction

The seven resistance loci that are effective against multiple races and/or consistently effective for field resistance were selected for exploring the frequencies of resistance-associated alleles present in STRUCTURE-based subpopulations (Table 6).

**Table 6:** Frequencies of resistance-associated loci to *Puccinia striiformis* f. sp. *tritici* (*Pst*) in STRUCTURE-based subpopulations

Name^a^	Chr^b^	CI (cM)^c^	RAF^d^	STRUCTURE-base subpopulation		
				One (African origin)	Two (European origin)	Three (Asian origin)
Seedling QTL						
*YrTtd*-*2BL.1*	2BL	138.3–142.5	0.14	0.38	0.04	0.08
*YrTtd*-*4AL.1*	4AL	160.7–164.9	0.11	0.38	0.04	0
*YrTtd*-4BL.1*	4BL	69.9–74.1	0.13	0.36	0.09	0
*YrTtd*-4BL.2*	4BL	116.7–120.9	0.31	0.42	0.09	0.55
*YrTtd*-*6AS.1*	6AS	9.2–13.4	0.17	0.64	0.01	0
*YrTtd*-7AS*	7AS	6.6–10.8	0.15	0.47	0.06	0
*YrTtd*-7AL.2*	7AL	132.2–136.4	0.53	0.49	0.88	0.04
Field QTL						
*QYrTtd*-*1A*	1A	50.9–55.1	0.59	0.05	0.77	1
*QYrTtd*-*1BS*	1BS	4.0–8.2	0.3	0.73	0.08	0
*QYrTtd*-*1BL*	1BL	103.9–108.1	0.18	0	0.15	0.35
*QYrTtd*-*2AS*	2AS	61.3–65.5	0.03	0	0.23	0
*QYrTtd*-*2AL*	2AL	105.6–109.8	0.37	0.07	0.31	0.63
*QYrTtd*-*2BS.1*	2BS	7.9–12.1	0.6	0.48	0.77	0.65
*QYrTtd*-*2BS.2*	2BS	74.7–78.9	0.72	0.73	0.92	0.65
*QYrTtd*-*3BS.1*	3BS	46.8–51.0	0.46	0	0.46	0.85
*QYrTtd*-*3BS.2*	3BS	75.0–79.2	0.61	0.75	0	0.63
*QYrTtd*-*6BS.1*	6BS	5.9–10.1	0.94	1	0.85	0.9
*QYrTtd*-*6BS.2*	6BS	16.2–20.4	0.32	0.68	0.38	0
*QYrTtd*-*7AL.1*	7AL	190.9–195.1	0.96	1	0.77	0.98
*QYrTtd*-*7AL.2*	7AL	198.7–202.9	0.83	0.98	0.23	0.87
*QYrTtd*-*7BL*	7BL	191.1–195.3	0.34	0.02	0.31	0.62

^a^ The loci followed by asterisks confer resistance both at the seedling stage in the greenhouse and adult stages in the field

^b^ Chromosome

^c^ Confidence interval of ±2.1 cM from the peak of the tag-SNP

^d^ Resistance allele frequency in the whole panel

The African subpopulation had higher frequencies of associated SNP alleles for *YrTtd*-*2BL.1*, *YrTtd*-*4AL.1*, *YrTtd*-*4BL.1*, *YrTtd*-*6AS.1* and *YrTtd*-*7AS*. The favorable alleles for the majority of resistance loci detected in the seedling tests in subpopulation two (European materials) and three (Asian materials) had lower frequencies than that of subpopulation one. Exceptions are *YrTtd*-*7AL.2* and *YrTtd*-*4BL.2*, whose frequencies of favorable SNP-associated alleles are much higher in subpopulation two and subpopulation three, respectively. The lower frequencies of resistance-associated alleles in accessions of subpopulation two and three might explain their lower levels of resistance to *Pst* races PSTv-14, PSTv-37, PSTv-40, PSTv-51 and PSTv-125 at the seedling tests (Table 2).

Observing the distribution of favorable alleles for the 14 field resistance QTL (Table 6), the favorable alleles of *QYrTtd*-*6BS.1* and *QYrTtd*-*7AL.1* were almost fixed in the entire panel. Additionally, in subpopulation one, *QYrTtd*-*1BS*, *QYrTtd*-*3BS.2*, *QYrTtd*-*6BS.2* and *QYrTtd*-*7AL.2* had higher associated SNP allele frequencies than the other subpopulations, but the favorable alleles for *QYrTtd*-*1BL, QYrTtd*-*2AS* and *QYrTtd*-*3BS.1* were absent. The European-subgroup (subpopulation two) possessed higher resistance-associated allele frequencies for *QYrTtd*-*2AS*, *QYrTtd*-*2BS.1* and *QYrTtd*-*2BS.2.* Subpopulation three collected from Asian countries contributed more resistance alleles for *QYrTtd*-*1A*, *QYrTtd*-*2AL*, *QYrTtd*-*3BS.1* and *QYrTtd*-*7BL.* However, no significant differences were identified for the mean BLUP_IT across all environments in three subpopulations. For the mean BLUP_SEV, subpopulation one was significantly (*P* < 0.05) different from subpopulation three, but not subpopulation two (Table 2).

## Discussion

### SNP genome properties and genetic diversity revealed by 90K SNP array

The rapid advancement in next-generation sequencing in the last few years has enabled the development of genome-wide SNP arrays for the polyploidy wheat genome. In this study, the currently available 90K SNP wheat array (Wang et al. 2014b) was used to genotype the cultivated emmer wheat accessions. In spite of ascertainment bias when using the 90K SNP array on cultivated emmer wheat and the presence of D-genome markers, we were still able to detect 7756 polymorphic SNPs after data quality filtering steps, of which 5106 were positioned on a tetraploid wheat consensus map. A total genome coverage of 2542 cM, with an average marker density of 2.0 SNP/cM and an average inter-marker distance of 0.5 cM/SNP were found in this population. The 4.2 cM confidence interval of loci contained over eight polymorphic SNPs suitable for GWAS, on average. Therefore, the wheat 90K SNP array provided sufficient polymorphic markers for this cultivated emmer wheat population to carry out genetic diversity, population structure, LD and association mapping analyses.

Although many populations have been assembled to study the genetic diversity of different species of wheat (Autrique et al. 1996; Barcaccia et al. 2002; Maccaferri et al. 2003; Zhang et al. 2010a), this is the first using the high-density 90K SNP array on cultivated emmer wheat. The accessions in this study were sampled from nearly all major emmer wheat growing regions, and comprehensively sampled gene pools of spring emmer wheat, as recently described in Badaeva et al. (2015). Therefore, information on genetic diversity and LD of this population can offer relevant guidelines for assembling association mapping populations and designing new breeding strategies for exploiting and utilizing cultivated emmer wheat genetic resources to improve durum and bread wheat cultivars.

The genome-wide 90K SNP markers revealed an average genetic diversity index of 0.2895, PIC of 0.2321 and MAF of 0.2168 for this cultivated emmer wheat population. These values are slightly elevated compared to previously reported estimates of cultivated durum and bread wheat populations also using genome-wide SNP markers. Other researchers reported a genetic diversity value of 0.2280 and PIC of 0.1888 among a worldwide germplasm collection of 150 durum wheat genotypes (Ren et al. 2013b), and PIC of 0.20 among 478 US and CIMMYT spring and winter bread wheat cultivars (Chao et al. 2010). The higher genetic diversity of cultivated emmer wheat may be attributed to its longer cultivation in a large range of eco-geographical conditions, a longer history of recombination and less domestication bottlenecks than the later-domesticated durum wheat and bread wheat (Zaharieva et al. 2010; Badaeva et al. 2015). Lower genetic diversity (0.1841) and PIC (0.1530) values were also reported in a collection of 200 wild emmer wheat lines from Israel and Turkey (Ren et al. 2013a), which may be explained by a more narrow range of geographical distribution of this wild emmer wheat panel than our cultivated emmer wheat panel sampled globally.

### Linkage disequilibrium in cultivated emmer wheat

The effectiveness of GWAS experiments heavily depends on LD decay rates regulated by the genetic distance between loci, the number of generations since it arose (Mackay and Powell 2007) and, consequently, the density and genome-wide coverage of the molecular polymorphisms used in the MTA tests. Based on 5106 SNP markers that yielded 1892,402 intra-chromosomal SNP pairs to calculate the LD *r* ^2^ values, the genome-wide *r* ^2^ values abruptly declined to 0.2 within 1 cM in this cultivated emmer wheat panel. Sela et al. (2014) observed a similar LD decay rate in a wild emmer wheat population. When compared with cultivated durum wheat and bread wheat, LD decayed more rapidly in this cultivated emmer wheat collection. Previous studies reported that the genome-wide LD *r* ^2^ decayed to 0.2 within a distance of 5–20 cM in the elite bread wheat populations (Chao et al. 2007; Naruoka et al. 2015; Zegeye et al. 2014; Zhang et al. 2010a). Long-range LD was found at locus pairs with an intra-chromosomal distance more than 50 cM using 70 SSR markers in durum wheat (Maccaferri et al. 2005). The faster LD decay rate observed in this panel indicates a higher mapping resolution is possible when conducting GWAS on cultivated emmer wheat than durum and bread wheat.

### Map-based comparison of the significant all-stage resistance loci with previously published *Yr* genes

In this emmer wheat panel, a total of 37 loci were significantly associated with all-stage resistance to *Pst* races detected in the seedling tests. *YrTtd*-*1AS*, *YrTtd*-*1BS*, *YrTtd*-*1BL*, *YrTtd*-*2BS*, *YrTtd*-*4AL.1*, *YrTtd*-*5AL.1*, *YrTtd*-*5BS.1*, *YrTtd*-*5BL.1*, *YrTtd*-*6BS*, *YrTtd*-*6BL*, *YrTtd*-*7AL.2* and *YrTtd*-*7BL* co-locate with resistance loci characterized from a GWAS study of elite durum wheat (Liu et al. 2017a) based on the tetraploid consensus map (Supplemental Table 5). Only *YrTtd*-*4AL.1*, *YrTtd*-*5BL.1* and *YrTtd*-*6BL* were associated with the same *Pst* races as these resistance loci tagged by IWB31333, IWB48863 and IWB55752, respectively, which were detected in the elite durum wheat panel. Haplotype analyses indicate that *YrTtd*-*6BL* and IWB55752 are likely to be the same loci because they are in high level of LD (*r* ^2^ = 0.8), but *YrTtd*-*4AL.1* and IWB31333 on chromosome 4AL, *YrTtd*-*5BL.1* and IWB48863 on chromosome 5BL are most likely different genes since the LD *r* ^2^ values between these two pairs of markers are less than 0.3.

Further map-based comparison of significant all-stage resistance loci with previously reported *Yr* genes were conducted by using the integrated genetic map (Maccaferri et al. 2015b, Supplemental Table 5). On chromosome arm 1BS, *YrTtd*-*1BS* mapped within the gene region of *Yr64* and very close to *Yr65*. *Yr64* and *Yr65* were resistant to all the tested races including PSTv-40, PST-100 (=PSTv-37) and PST-127 (=PSTv-14) (Cheng et al. 2014) used in the present study. However, *YrTtd*-*1BS* was significantly associated with resistance to PSTv-40. This result illustrated that *YrTtd*-*1BS* is likely neither *Yr64* nor *Yr65*, and indicated this region of chromosome 1B harbors several alleles or a cluster of *Yr* genes (Cheng et al. 2014).

*YrTtd*-*2BL.1* spanned the confidence interval of the all-stage resistance gene *Yr53*. *Yr53* was characterized from the durum wheat ‘PI 480148’, which was originally collected from Shewa, Ethiopia, in 1973, and conferred resistance to nine tested US *Pst* races including PST-100 and PST-127 (equivalent to PSTv-14 and PSTv-37) (Xu et al. 2013; Wan and Chen 2014). In the current study, *YrTtd*-*2BL.1* was associated with resistance to PSTv-37, PSTv-40 and PSTv-51, but not PSTv-14. Therefore, it is possible that *YrTtd*-*2BL.1* is not *Yr53*.

On chromosome 4A, *YrTtd*-*4AL.1* resided in the confidence interval of resistance gene *Yr60* (Herrera-Foessel et al. 2015)*. YrTtd*-*4AL.1* was significantly associated with response to race PSTv-37, PSTv-40 and PSTv-51 in the current panel, whereas *Yr60* conferred a moderate level of resistance to *Pst* isolate Mex96.11. It is possible that *YrTtd*-*4AL.1* represents the same resistance gene as *Yr60*, but seedling evaluation for both donor plants with the same *Pst* races and/or allelism testing are needed to further investigate their relationships. Moreover, Maccaferri et al. (2015b) reported that IWA1034 was significantly associated with APR in multiple environments, but was not detected by GWAS analysis of resistance to races PSTv-4, PSTv-14, PSTv-37 and PSTv-40 in a worldwide collection of hexaploid spring bread wheat. Since two APR QTL (Chen et al. 2012; Prins et al. 2011) have already been identified in this chromosome region, IWA1034 may be closely linked with both all-stage resistance genes and APR genes.

In the proximal region of chromosome arm 4BL, *YrTtd*-*4BL.1* was significantly associated with responses to PSTv-37 and PSTv-125 in the seedling tests and field responses in MTV14 and SPM15 in this cultivated emmer panel, but not associated with responses to PSTv-40. *YrTtd*-*4BL.1* overlapped with *Yr50* (Liu et al. 2013) and *Yr62* (Lu et al. 2014). However, *YrTtd*-*4BL.1* is most likely not *Yr62*; *Yr62* is an APR gene. *Yr50* confers race-specific all-stage resistance, putatively derived from *Thinopyrum intermedium* in Chinese winter wheat cultivar ‘CH223’. Lu et al. (2014) conducted the seedling test on ‘CH223’ and confirmed *Yr50* was highly resistant to PST-116 (=PSTv-40) and PST-127 (=PSTv-14). Given the wild relative origin and the different reactions of *YrTtd*-*4BL.1* and *Yr50* to the same *Pst* races, it is unlikely that *YrTtd*-*4BL.1* and *Yr50* represent the same gene.

The other 22 significant resistance loci detected in the seedling tests in this GWAS study appeared to be novel since they are located on the chromosome regions where no race-specific all-stage resistance genes have been mapped before, apart from IWB63861 (*YrTtd*-*6AS.*1) because map position for this marker is unknown in the hexaploid integrated map.

*YrTtd*-*4BS* and *YrTtd*-*5BL.2* were only significantly associated with the least virulent race PSTv-18. Therefore, *YrTtd*-*4BS* and *YrTtd*-*5BL.2* should be considered relic loci with limited effectiveness against contemporary *Pst* populations. Four resistance loci (*YrTtd*-*2BS*, *YrTtd*-*2BL.3*, *YrTtd*-*4BL.2* and *YrTtd*-*7AS*) on chromosomes 2BS, 2BL, 4BL and 7AS were significantly associated with resistance with Italian race PSTv-125. Among them, *YrTtd*-*2BS* and *YrTtd*-*2BL.3* were exclusively associated with resistance to PSTv-125. On the other hand, *YrTtd*-*4BL.2* and *YrTtd*-*7AS* were more broadly effective than other resistance loci detected in this study because they were detected with three or more *Pst* races in the seedling tests and in fields of multiple environments. Hence, *YrTtd*-*4BL.2* and *YrTtd*-*7AS* are effective all-stage resistance genes to be introgressed into adapted durum and bread wheat accessions.

In addition, all-stage resistance genes *Yr5* and *Yr15* are known to be effective against all *Pst* races identified so far in the United States (Murphy et al. 2009; Wan and Chen 2014) and have been incorporated into numerous bread and durum wheat cultivars (Peng et al. 2000; Sun et al. 1997). In this cultivated emmer wheat population, no significant associations were detected close to the putative positions of *Yr5* and *Yr15*. In addition, two flanking markers KASP_IWA6121 and KASP_IWA4096 (Naruoka et al. 2016) and a tightly linked SSR marker barc8 (Yaniv et al. 2015) were used to screen the panel for the presence/absence of *Yr5* and *Yr15*, respectively; none of the accessions carried the expected marker alleles for *Yr5* or *Yr15*.

### Field resistance loci and their relations to previously reported stripe rust QTL

It is not surprising that four all-stage resistance loci (*YrTtd*-*4BL.1*, *YrTtd*-*4BL.2*, *YrTtd*-*7AS* and *YrTtd*-*7AL.2*) conferred resistance to stripe rust in the field because *Pst* race PSTv-37 that was used in the seedling test prevailed among five field screening nurseries except CLF15, and *YrTtd*-*4BL.1*, *YrTtd*-*4BL.2* and *YrTtd*-*7AS* were detected to be associated with resistance to PSTv-37.

A total of 14 loci that were significantly associated with adult plant response in the field but not seedling response were characterized in this emmer wheat population, but only *QYrTtd*-*3BS.1* showed consistent associations with field resistance in multiple screening nurseries at the significance level of *P* < 0.001. When applying a less stringent significance level of *P* < 0.005, *QYrTtd*-*1BL*, *QYrTtd*-*2AL*, *QYrTtd*-*2BS.2*, *QYrTtd*-*3BS.2* and *QYrTtd*-*6BS.1* were significantly associated with resistance in at least two environments. The other eight loci that showed significant associations within single environments should be deployed cautiously since they are unstable in different environments and susceptible to some *Pst* races.

Comparing the APR loci putatively identified in this study with those identified in an elite durum wheat GWAS panel (Liu et al. 2017a), *QYrTtd*-*2AL* (tagged by IWB4635) mapped within the confidence interval of *QYrdurum*-*2AL* (tagged by IWA1008). However, most of the SNP markers in the haplotype of *QYrdurum*-*2AL* were not polymorphic in the emmer wheat panel which made it difficult to establish the relationship between *QYrTtd*-*2AL* and *QYrdurum*-*2AL*.

Further map-based comparisons of significant APR loci in this study with known APR loci were performed on the basis of the consensus map generated by Maccaferri et al. (2015b). Seven additional resistance loci in this emmer wheat panel overlapped with known resistance QTL (Supplemental Table 5). *QYrTtd*-*1A*, spanning the centromere of chromosome 1A, maps to the confidence interval of APR QTL *QYr.sun*-*1A*. *QYr.sun*-*1A* was derived from the Australia cultivar ‘Janz’ (Bariana et al. 2010), and had race-specific resistance to *Pst* race 110 E143A+ but susceptibility to 134 E16A + . *QYrTtd*-*1A* is likely race-specific since it is identified at a significance level of *P* < 0.005 in only one environment (CLF15). Allelism testing would be needed to determine the relationship of *QYrTtd*-*1A* and *QYr.sun*-*1A*.

Previously reported APR QTL, *QYr.cau*-*1BS*, mapped to the confidence interval of *QYrTtd*-*1BS* on chromosome arm 1BS. *QYr.cau*-*1BS* was characterized from the wheat breeding line ‘AQ24788-83’ that is the progeny of a double cross consisting of four stripe rust-resistant Chinese winter wheat landraces (Quan et al. 2013). However, *QYr.cau*-*1BS* contributed to prolonged latency for the appearance of uredinia but not reduced IT and SEV, hence, *QYr.cau*-*1BS* and *QYrTtd*-*1BS* may not be the same QTL.

On the short arm of chromosome 2B, two loci detected in the field tests in this GWAS overlapped with known APR QTL. *QYr.inra*-*2BS* derived from ‘Renan’ (Dedryver et al. 2009) and *QYrst.orr*-*2BS.1* derived from ‘Stephens’ (Vazquez et al. 2012) mapped in the same region where *QYrTtd*-*2BS.1* was identified in the current study. ‘Stephens’ was a soft white winter cultivar grown in the Pacific Northwest, USA and has a combination of all-stage and APR resistance. Stephens conferred resistance in the PNW region for more than 30 years and in recent years this resistance has been mostly been explained by APR loci. Five APR loci have been detected from ‘Stephens’ by Vazquez et al. (2012). *QYrst.orr*-*2BS.1*, peaked at wPt-5738, was a minor QTL that was unstable across environments. Similarly, *QYrTtd*-*2BS.1* in the current study was only detected in CLF15 but not the other environments. *QYrTtd*-*2BS.2* falls into the confidence intervals of six previously reported APR regions including *QYrlo.wpg*-*2BS_Louise* (Carter et al. 2009), *QYrid.ui*-*2B.2_*IDO444 (Chen et al. 2012), *QYr.caas*-*2BS_Pingyuan 50* (Lan et al. 2010), *QYr*-*2B_Opata 85* (Boukhatem et al. 2002), *QYr.tam*-*2BL_TAM111* (Basnet et al. 2014) and *QYr.ucw*-*2B_UC1110* (Lowe et al. 2011). Additionally, three race-specific all-stage resistant genes *YrKK* (Li et al. 2013), *Yr41* (Luo et al. 2008) and *Yr27* (McDonald et al. 2004) were also clustered here. This region is very close to the centromere of chromosome 2B, where recombination frequencies are greatly reduced. Therefore, allelism tests for evaluation of the complex relationships among these *Yr* loci are difficult in this chromosome region.

The emmer wheat-derived pleiotropic APR locus *Sr2/Yr30* is located on the distal part of chromosome 3BS. No MTAs in this emmer wheat panel were identified on the genetic region of *Sr2/Yr30.* Profiling with the diagnostic marker wMAS000005 of *Sr2/Yr30*, only four accessions (“MG5287/1” from Iran, “MG5297/1” from Bulgaria, “MG5442” from the United States and “MG15518” from Syria) were detected to carry this particular allele in this panel.

A haplotype of five SNPs (IWB124, IWB5813, IWB25636, IWB32812 and IWB50708) was located near the centromeric region of chromosome arm 3BS and was designated as *QYrTtd*-*3BS.2* in our study. Results of haplotype analysis showed a high level of LD among these SNPs with pairwise *r* ^2^ equal to 0.90. Based on the integrated map, the confidence interval of *QYrTtd*-*3BS.2* overlaps with the region of *QYrco.wpg*-*3BS.2* which represents a minor APR locus detected from the soft white winter wheat cultivar ‘Brundage’ (Case et al. 2014).

On the long arm of chromosome 7A, *QYrTtd*-*7AL.2* resided within the QTL region of *QYr.cim*-*7AL* that was identified from a stripe rust susceptible cultivar ‘Avocet’ by Rosewarne et al. (2012). *QYr.cim*-*7AL*, flanked by marker wPt-2260 and wPt-2501, is a minor APR locus in cultivar ‘Avocet’, that showed high susceptibility to stripe rust in the field. But *QYrTtd*-*7AL.2* identified in our study is a major effect QTL that explained 9.1% of phenotypic variation for IT in SPM15.

*QYrTtd*-*7BL* (tagged by IWB72249) detected on chromosome arm 7BL in this study overlapped with the marker interval between wgp5175 and barc32, designated as *Yr59* by Zhou et al. (2014). *Yr59* was characterized from the Iranian spring wheat landrace ‘PI 178759’ and confers HTAP resistance. Allelism testing would be needed to verify whether *QYrTtd*-*7BL* is *Yr59* or not.

### Sources of resistance and susceptibility to *Pst* races

Breeding for *Pst* resistance in cultivated durum and bread wheat continues to be impeded by the lack of resistance sources effective against contemporary *Pst* races in elite genetic backgrounds. The relatives of these wheat species are promising resistance sources to provide various resistance genes (Uauy et al. 2005; Yin et al. 2006; Marais et al. 2009; Liu et al. 2013). In the current study, we evaluated the reactions of 176 cultivated emmer wheat accessions to four contemporary *Pst* races of the United States (PSTv-14, PSTv-37, PSTv-40 and PSTv-51) and one Italian race (PSTv-125) at the seedling stage. About 9, 10, 14, 4 and 8% of accessions were resistant to PSTv-14, PSTv-37, PSTv-40, PSTv-51 and PSTv-125, respectively.

Among them, accessions “IDG392”, “MG5346/1”, “MG5362”, “MG5530” from Ethiopia and “MG5312” from Afghanistan were resistant to all races in seedling tests and displayed moderate to high levels of resistance in adult plants across six environments. Accordingly, these accessions may be good donor parents for wheat breeding. Conversely, 14 accessions from Italy, the United Kingdom, Spain, Romania, Germany, Yugoslavia and Iran (Supplemental File 1), were identified as ideally susceptible parents for geneticists to construct bi-parental mapping populations. These accessions were highly susceptible to all tested races at the seedling stage. At the adult stages, 13 of them exhibited winter habit or very late heading dates. The remaining one accession (“MG29292” from former Yugoslavia) was susceptible in field nurseries and would be a good susceptible parent for future studies.

In this cultivated emmer population, four accessions (“MG5287/1” from Iran, “MG5297/1” from Bulgaria, “MG5442” from the United States, and “MG15518” from Syria) were detected to carry the resistance allele of *Sr2*/*Yr30* as well as resistance-associated alleles for *QYrTtd*-*1A*, *QYrTtd*-*2AL*, *QYrTtd*-*2BS.1*, *QYrTtd*-*2BS.2*, *QYrTtd*-*3BS.1*, *QYrTtd*-*3BS.2*, *QYrTtd*-*6BS.1* and *QYrTtd*-*7AL.1.* These accessions show moderate to high resistance at the adult stages but susceptibility to most tested races at the seedling stage. Hence, they may serve as donor parents of APR for tetraploid wheat breeding.

## Conclusion

Cultivated emmer wheat, as an important genetic resource for durum and bread wheat improvement, has attracted the attention of wheat researchers in recent years. Our study reports the presence of valuable genetic variation for multiple *Pst* races in seedling tests and in field nurseries. The cultivated emmer wheat accessions that showed all-stage resistance or APR could be crossed with adapted durum and bread cultivars to enhance stripe rust resistance, particularly those accessions carrying improved resistance alleles at novel loci. High-density, whole-genome SNP markers revealed a high level of genetic diversity and relatively fast LD decay, indicating that cultivated emmer wheat is suitable for GWAS studies aimed at directly identifying MTAs closely linked to the causal loci. In the GWAS analyses, 37 loci significantly associated with all-stage resistance and 14 loci that were associated with field resistance were found. More than half of these were mapped to chromosome regions where no stripe rust resistance genes have been reported before, indicating that a large reservoir of useful alleles currently under-exploited in the modern breeding germplasm is present in domesticated emmer. This result highlights the presence of novel *Pst* resistance loci in cultivated emmer wheat that could be pyramided into durum and bread wheat cultivars by recurrent selection or marker-assisted selection. Finally, the relatively large number and small phenotypic effects of loci detected in this cultivated emmer wheat population demonstrated the complex genetic basis of stripe rust resistance in wheat, suggesting that genomic selection might be a more comprehensive strategy towards accumulation of beneficial alleles in breeding and pre-breeding populations than conventional marker-assisted selection.

### Author contribution statement

WL managed and performed all the experiments, analyzed the data, interpreted the results, and wrote the manuscript. MP supervised the experiments, advised data analyses and revised the manuscript. MM advised data analyses and revised the manuscript. XC, RT, DP and GL provided materials and revised the manuscript.

## Electronic supplementary material

Below is the link to the electronic supplementary material.

**Supplemental File 1** Accession names and origins of 176 cultivated emmer wheat and their reactions to *Puccinia striiformis* f. sp*. tritici* (*Pst*) at the seedling and adult stages (XLSX 33 kb)

**Supplemental Table** **1** Virulence/avirluence formula of six complementary *Puccinia striiformis* f. sp. *tritici* (*Pst*) races used for seedling evaluations (DOCX 66 kb)

**Supplemental Table** **2** Primer sequences of diagnostic markers for *Yr5*, *Yr15* and *Sr2/Yr30* used for profiling the cultivated emmer wheat panel (DOCX 49 kb)

**Supplemental Table** **3** Bayesian information criterion (BIC) values of different genome-wide associate models (DOCX 90 kb)

**Supplemental Table** **4** Marker distribution, minor allele frequency (MAF), number of alleles, genetic diversity and polymorphism information content (PIC) values in the cultivated emmer wheat population collected from worldwide (DOCX 77 kb)

**Supplemental Table** **5** Relations of significant race-specific seedling and field resistance loci to previously published *Yr* genes/QTL based on map positions of the integrated map (DOCX 100 kb)
