# Supplementary material for: Genome-wide association mapping reveals a rich genetic architecture of stripe rust resistance loci in emmer wheat (Triticum turgidum ssp. dicoccum)
Source: Theor Appl Genet. 2017 Aug 2;130(11):2249–70. doi: 10.1007/s00122-017-2957-6 (PMC5641275; doi:10.1007/s00122-017-2957-6)
Supplement: Supplementary file 2 — Supplemental Table 1 Virulence/avirluence formula of six complementary Puccinia striiformis f. sp. tritici (Pst) races used for seedling evaluations (DOCX 66 kb) [file 122_2017_2957_MOESM2_ESM.docx]

**Supplemental Table 1.** Virulence/avirluence formula of six complementary *Puccinia striiformis* f. sp. *tritici* (*Pst*) races used for seedling evaluations.

| ***Pst* race (isolate)^a^** | **Virulence/ Avirulence formula^b^** |
| --- | --- |
| PSTv-14 (11-116-NG) | ***1,6,7,8,9,17,27,43,44,Tr1,Exp2,Tye***/*5,10,15,24,32,SP* |
| PSTv-18 (11-281-NG) | /*1,5,6,7,8,9,10,15,17,24,27,32,43,44,SP,Tr1,Exp2,Tye* |
| PSTv-37 (12-114-NG) | ***6,7,8,9,17,27,43,44,Tr1,Exp2***/*1,5,10,15,24,32,SP,Tye* |
| PSTv-40 (09-78) | ***6,7,8,9,10,24,27,32,43,44,Tr1,Exp2***/*1,5,15,17,SP,Tye* |
| PSTv-51 (11-366) | ***1,6,7,8,9,10,17,24,27,32,43,44,SP,Tr1,Exp2,Tye***/*5,15* |
| PSTv-125 (IT14-13) | ***1,6,7,9,43,44,SP,Exp2,Tye***/*5,9,10,15,17,24,27,32,Tr1* |

^a^ 11-116-NG, 11-281-NG, 12-114-NG, 09-78, 11-366 and IT14-13 are the isolate numbers of PSTv-14, PSTv-18, PSTv-37, PSTv-40, PSTv-51 and PSTv-125, respectively.

^b^ The virulence/avirulence formula was developed based on the responses of the 18 *Yr* near isogenic lines in the ‘Avocet S’ background to *Pst*: *1* = AvSYr1NIL (*Yr1*); *5* = AvSYr5NIL (*Yr5*); *6* = AvSYr6NIL (*Yr6*); *7* = AvSYr7NIL (*Yr7*); *8* = AvSYr8NIL (*Yr8*); *9* = AvSYr9NIL (*Yr9*); *10* = AvSYr10NIL (*Yr1*0); *17* = AvSYr17NIL (*Yr17*); *24* = AvSYr24NIL (*Yr24*); *27* = AvSYr27NIL (*Yr27*); *32* = AvSYr32NIL (*Yr32*); *43* = AvS/IDO377s (F3-41-1) (*Yr43*); *44* = AvS/Zak (1-1-35-line1) (*Yr44*); *SP* = AvSYrSPNIL (*YrSP*); *Tr1* = AvSYrTres1NIL (*YrTr1*); *Exp2* = AvS/Exp 1/1-1 Line 74 (*YrExp2*); *Tye* = Tyee (*YrTye*) (Wan and Chen, 2014).
