# Supplementary material for: Genome-wide association mapping reveals a rich genetic architecture of stripe rust resistance loci in emmer wheat (Triticum turgidum ssp. dicoccum)
Source: Theor Appl Genet. 2017 Aug 2;130(11):2249–70. doi: 10.1007/s00122-017-2957-6 (PMC5641275; doi:10.1007/s00122-017-2957-6)
Supplement: Supplementary file 3 — Supplemental Table 2 Primer sequences of diagnostic markers for Yr5, Yr15 and Sr2/Yr30 used for profiling the cultivated emmer wheat panel (DOCX 49 kb) [file 122_2017_2957_MOESM3_ESM.docx]

**Supplemental Table 2.** Primer sequences of diagnostic markers for *Yr5*, *Yr15* and *Sr2/Yr30* used for profiling the cultivated emmer wheat panel.

|  | **Marker** | | **Primer sequence (5'-3')** | |
| --- | --- | --- | --- | --- |
| **Gene** | **Name** | **Type** | **Forward** | **Reverse** |
| *Yr5* | IWA6121 | KASP | CCAGTGCTGGTGAAAAGCGTGA[T/C]^a^ | CCAACAAAGATTGTATAGTCCGGGGTA |
|  | IWA4096 | KASP | GCCCAGCCTGTACACCC [A/G] | TTTGATCTGAGCTGTAAATGTGTCA |
| *Yr15* | barc8 | SSR | GCGGGAATCATGCATAGGAAAACAGAA | GCGGGGGCGAAACATACACATAAAAACA |
| *Yr30/Sr2* | wMAS000005 | KASP | GTGCGAGACATCCAACACTCA [C/T] | CTCAAATGGTCGAGCACAAGCTCTA |

^a^ SNP identity of KASP markers are exhibited by bracket.
