# Supplementary material for: Genome-wide association mapping reveals a rich genetic architecture of stripe rust resistance loci in emmer wheat (Triticum turgidum ssp. dicoccum)
Source: Theor Appl Genet. 2017 Aug 2;130(11):2249–70. doi: 10.1007/s00122-017-2957-6 (PMC5641275; doi:10.1007/s00122-017-2957-6)
Supplement: Supplementary file 4 — Supplemental Table 3 Bayesian information criterion (BIC) values of different genome-wide associate models (DOCX 90 kb) [file 122_2017_2957_MOESM4_ESM.docx]

**Supplemental Table 3.** Bayesian information criterion (BIC) values of different genome-wide associate models.

|  | **Seedling** | | |
| --- | --- | --- | --- |
| **Isolate** | ***Q* GLM** | ***K* MLM** | ***Q*+*K* MLM** |
| PSTv-14 | -358.11 | -328.87 | -332.99 |
| PSTv-18 | -432.33 | -366.74 | -371.36 |
| PSTv-37 | -384.35 | -336.97 | -341.15 |
| PSTv-40 | -407.52 | -372.17 | -374.79 |
| PSTv-51 | -341.84 | -320.71 | -324.66 |
| PSTv-125 | -394.95 | -367.51 | -372.67 |
|  | **Field** | | |
| **Environment** | ***Q* GLM** | ***K* MLM** | ***Q*+*K* MLM** |
| MTV14_IT | -254.06 | -238.34 | -240.78 |
| MTV14_SEV | -550.13 | -534.85 | -536.99 |
| SPM14_IT | -246.28 | -233.43 | -235.78 |
| SPM14_SEV | -488.19 | -475.66 | -477.99 |
| WHT14_IT | -192.65 | -182.81 | -185.09 |
| WHT14_SEV | -481.78 | -461.29 | -463.58 |
| MTV15_IT | -238.5 | -224.16 | -226.34 |
| MTV15_SEV | -531.05 | -517.72 | -520.05 |
| CLF15_IT | -261.82 | -255.12 | -257.18 |
| CLF15_SEV | -514.74 | -501.28 | -502.87 |
| SPM15_IT | -236.57 | -223.01 | -225.17 |
| SPM15_SEV | -477.72 | -467.94 | -470.19 |
| BLUP_IT | -216.03 | -198.37 | -200.57 |
| BLUP_SEV | -486.68 | -462.35 | -464.69 |

***Q* GLM**: the general linear model (GLM) corrected for population structure using STRUCTURE membership coefficients (*Q* matrix); ***K* MLM**: MLM corrected for kinship identity-by-state allele-sharing matrix (*K* matrix); ***Q*+*K* MLM**: MLM with *Q* and *K* matrix as covariates. **IT**: infection type; **SEV**: disease severity; **MTV14**: Mount Vernon in 2014; **SPM14**: Spillman farm in 2014; **WHT14**: Whitlow farm in 2014; **MTV15**: Mount Vernon in 2015; **CLF15**: Central Ferry in 2015; **SPM15**: Spillman farm in 2015; **BLUP**: best linear unbiased predictor. The optimal GWAS models for seedling and field resistance were highlighted in red because larger BIC is better (Lipka et al. 2012).
