# Supplementary material for: Genome-wide association mapping reveals a rich genetic architecture of stripe rust resistance loci in emmer wheat (Triticum turgidum ssp. dicoccum)
Source: Theor Appl Genet. 2017 Aug 2;130(11):2249–70. doi: 10.1007/s00122-017-2957-6 (PMC5641275; doi:10.1007/s00122-017-2957-6)
Supplement: Supplementary file 5 — Supplemental Table 4 Marker distribution, minor allele frequency (MAF), number of alleles, genetic diversity and polymorphism information content (PIC) values in the cultivated emmer wheat population collected from worldwide (DOCX 77 kb) [file 122_2017_2957_MOESM5_ESM.docx]

**Supplemental Table 4.** Marker distribution, minor allele frequency (MAF), number of alleles, genetic diversity and polymorphism information content (PIC) values in the cultivated emmer wheat population collected from worldwide.

| **Chromosome** | **No. of SNP markers** | **No. of polymorphic marker** | **MAF** | **No. of alleles detected** | **Mean value of genetic diversity** | **Mean PIC value** |
| --- | --- | --- | --- | --- | --- | --- |
| A genome |  |  |  |  |  |  |
| 1A | 1,326 | 324 (24.43%) | 0.2209 | 1.9472 | 0.2965 | 0.2377 |
| 2A | 1,493 | 327 (21.90%) | 0.1876 | 1.9183 | 0.2541 | 0.2051 |
| 3A | 1,146 | 243 (21.20%) | 0.1966 | 1.9206 | 0.2656 | 0.2141 |
| 4A | 1,022 | 292 (28.57%) | 0.2306 | 1.9325 | 0.3060 | 0.2445 |
| 5A | 1,137 | 279 (24.54%) | 0.2198 | 1.9235 | 0.2926 | 0.2340 |
| 6A | 1,324 | 268 (20.24%) | 0.1998 | 1.9298 | 0.2710 | 0.2195 |
| 7A | 1,542 | 364 (23.61%) | 0.2123 | 1.9222 | 0.2837 | 0.2275 |
| Subtotal/Mean | 8,990 | 2,097 (23.33%) | 0.2087 | 1.9275 | 0.2802 | 0.2251 |
| B genome |  |  |  |  |  |  |
| 1B | 1,937 | 540 (27.88%) | 0.2234 | 1.9365 | 0.2974 | 0.2378 |
| 2B | 2,291 | 564 (24.62%) | 0.2240 | 1.9446 | 0.2970 | 0.2377 |
| 3B | 1,549 | 399 (25.76%) | 0.2312 | 1.9477 | 0.3067 | 0.2447 |
| 4B | 1,026 | 307 (29.92%) | 0.2146 | 1.9113 | 0.2853 | 0.2281 |
| 5B | 1,696 | 429 (25.29%) | 0.2132 | 1.9292 | 0.2847 | 0.2287 |
| 6B | 1,576 | 348 (22.08%) | 0.2304 | 1.9194 | 0.3063 | 0.2449 |
| 7B | 1,665 | 422 (25.35%) | 0.2225 | 1.9237 | 0.2965 | 0.2373 |
| Subtotal/Mean | 11,740 | 3,009 (25.63%) | 0.2231 | 1.9322 | 0.2967 | 0.2374 |
| Homoeologous |  |  |  |  |  |  |
| 1 | 3,263 | 864 (26.48%) | 0.2224 | 1.9409 | 0.2970 | 0.2378 |
| 2 | 3,784 | 891 (23.55%) | 0.2097 | 1.9342 | 0.2801 | 0.2248 |
| 3 | 2,695 | 642 (23.82%) | 0.2165 | 1.9362 | 0.2893 | 0.2317 |
| 4 | 2,048 | 599 (29.25%) | 0.2226 | 1.9219 | 0.2956 | 0.2363 |
| 5 | 2,833 | 708 (24.99%) | 0.2158 | 1.9269 | 0.2879 | 0.2309 |
| 6 | 2,900 | 616 (21.24%) | 0.2164 | 1.9241 | 0.2902 | 0.2333 |
| 7 | 3,207 | 786 (24.51%) | 0.2176 | 1.9230 | 0.2904 | 0.2326 |
| Total/Grand mean | 20,730 | 5,106 (24.63%) | 0.2168 | 1.9301 | 0.2895 | 0.2321 |
