# Supplementary material for: Genome-wide association mapping reveals a rich genetic architecture of stripe rust resistance loci in emmer wheat (Triticum turgidum ssp. dicoccum)
Source: Theor Appl Genet. 2017 Aug 2;130(11):2249–70. doi: 10.1007/s00122-017-2957-6 (PMC5641275; doi:10.1007/s00122-017-2957-6)
Supplement: Supplementary file 6 — Supplemental Table 5 Relations of significant race-specific seedling and field resistance loci to previously published Yr genes/QTL based on map positions of the integrated map (DOCX 100 kb) [file 122_2017_2957_MOESM6_ESM.docx]

**Supplemental Table 5.** Relations of significant race-specific seedling and field resistance loci to previously published *Yr* genes/ QTL based on map positions of the integrated map.

|  |  | **Tetraploid^a^** | |  | **Integrated map^b^** | | |  |
| --- | --- | --- | --- | --- | --- | --- | --- | --- |
| **Locus** | **Tag-SNP** | **Chrom** | **CI (cM)** |  | **CI (cM)** | **Overlapping Yr genes/QTL** | **Reference** | **Associated SNP^c^** |
| *YrTtd-1AS* | IWB22778 | 1AS | 17.7-21.9 |  | 24.5-28.7 | IWB3519 | Liu et al. 2016 | - |
| *QYrTtd-1A* | IWA1279 | 1A | 50.9-55.1 |  | 68.6-72.8 | *QYr.sun-1A_Janz* | Bariana et al. 2010 | - |
| *YrTtd-1AL* | IWB27332 | 1AL | 86.2-90.4 |  | 97.7-101.9 | - | - | - |
| *QYrTtd-1BS* | IWB50501 | 1BS | 4.0-8.2 |  | 16.1-20.3 | *QYr.cau-1BS_AQ24788-53* | Quan et al. 2013 | - |
| *YrTtd-1BS* | IWB47025 | 1BS | 35.0-39.2 |  | 58.0-62.2 | *Yr64, Yrdurum-1BS.1, YrEDWL-1BS.1* | Cheng et al. 2014; Liu et al. 2017 | IWB14377, IWB47026, IWB49800, IWB62417, IWB64056 |
| *YrTtd-1BL* | IWB35698 | 1BL | 81.8-86.0 |  | 106.2-110.4 | IWB36872 | Liu et al. 2016 | - |
| *QYrTtd-1BL* | IWB69464 | 1BL | 103.9-108.1 |  | 117.3-121.5 | - | - | - |
| *QYrTtd-2AS* | IWB1046 | 2AS | 61.3-65.5 |  | 65.4-69.6 | - | - | - |
| *QYrTtd-2AL* | IWB4635 | 2AL | 105.6-109.8 |  | 82.7-86.9 | *QYrdurum-2AL* | Liu et al. 2016 | - |
| *YrTtd-2AL* | IWB67229 | 2AL | 160.1-164.3 |  | 174.4-178.6 | - | - | - |
| *QYrTtd-2BS.1* | IWB40673 | 2BS | 7.9-12.1 |  | 34.5-38.7 | *QYr.inra-2BS_Renan, QYrst.orr-2BS.1_Stephens* | Dedryver et al. 2009; Vazquez et al. 2012 | - |
| *YrTtd-2BS* | IWB7081 | 2BS | 25.6-29.8 |  | 59.2-63.4 | IWB32451 | Liu et al. 2016 | - |
| *QYrTtd-2BS.2* | IWB39220 | 2BS | 74.7-78.9 |  | 130.2-134.4 | *QYrlo.wpg-2BS_Louise, QYrid.ui2B.2_IDO444, QYr.caas-2BS_Pingyuan 50, QYr-2B_Opata 85, QYr.tam-2BL_TAM111, QYr.ucw-2B_UC1110* | Carter et al. 2009; Chen et al. 2012; Lan et al. 2010; Boukhatem et al. 2002; Basnet et al. 2014; Lowe et al. 2011 | - |
| *YrTtd-2BL.1* | IWB48012 | 2BL | 138.3-142.5 |  | 224.9-229.1 | *Yr53* | Xu et al. 2013 | - |
| *YrTtd-2BL.2* | IWB59983 | 2BL | 163.8-168.0 |  | 269.9-274.1 | - | - | - |
| *YrTtd-3AL* | IWB71901 | 3AL | 108.4-112.6 |  | 127.9-132.1 | - | - | IWB70903, IWB70904 |
| *QYrTtd-3BS.1* | IWB63252 | 3BS | 46.8-51.0 |  | 71.8-76.0 | - | - | - |
| *QYrTtd-3BS.2* | IWB124 | 3BS | 75.0-79.2 |  | 83.9-88.1 | *QYrco.wpg-3BS.2_Brundage* | Case et al. 2014 | IWA5813, IWB25636, IWB32812,IWB50708 |
| *YrTtd-3BS.1* | IWA7905 | 3BS | 84.3-88.5 |  | 90.4-94.6 | - | - | IWB4227, IWB63776 |
| *YrTtd-3BL.1* | IWB37522 | 3BL | 95.1--99.3 |  | 97.6-101.8 | - | - | IWB39508, IWA6510 |
| *YrTtd-3BL.2* | IWB59536 | 3BL | 147.8-152.0 |  | 144.7-148.9 | - | - | - |
| *YrTtd-3BL.3* | IWB10521 | 3BL | 185.5-189.7 |  | 179.9-184.1 | - | - | - |
| *YrTtd-4AS* | IWB55738 | 4AS | 23.1-27.3 |  | 26.9-31.1 | - | - | - |
| *YrTtd-4AL.1* | IWA1034 | 4AL | 160.7-164.9 |  | 179.7-183.9 | *Yr60*, IWB31333 | Herrera-Foessel et al. 2015, Liu et al. 2016 | - |
| *YrTtd-4BS* | IWB56078 | 4BS | 30.8-35.0 |  | 42.2-46.4 | - | - | - |
| *YrTtd-4BL.1* | IWA1641 | 4BL | 69.9-74.1 |  | 75.9-80.1 | *Yr50, Yr62* | Liu et al. 2013; Lu et al. 2014 | - |
| *YrTtd-4BL.2* | IWB67499 | 4BL | 116.7-120.9 |  | 123.1-127.3 | - | - | - |
| *YrTtd-5A* | IWB46475 | 5A | 61.7-65.9 |  | 67.3-71.5 | - | - | - |
| *YrTtd-5AL.1* | IWA1829 | 5AL | 140.4-144.6 |  | 130.9-135.1 | IWB73502 | Liu et al. 2016 | - |
| *YrTtd-5AL.2* | IWB67141 | 5AL | 181.1-185.3 |  | 177.9-182.1 | - | - | - |
| *YrTtd-5BS.1* | IWB10728 | 5BS | 36.1-40.3 |  | 45.6-49.8 | IWA2220 | Liu et al. 2016 | IWB9675 |
| *YrTtd-5BS.2* | IWB66991 | 5BS | 41.9-46.1 |  | 57.2-61.4 | - | - | - |
| *YrTtd-5BL.1* | IWB40681 | 5BL | 72.7-76.9 |  | 101.6-105.8 | IWB48863 | Liu et al. 2016 | - |
| *YrTtd-5BL.2* | IWA7733 | 5BL | 122.8-127.0 |  | 157.9-162.1 | - | - | - |
| *YrTtd-6AS.1* | IWB63861 | 6AS | 9.2-13.4 |  | - | - | - | - |
| *YrTtd-6AS.2* | IWB63758 | 6AS | 19.3-23.5 |  | 43.2-47.4 | - | - | - |
| *YrTtd-6AL.1* | IWB9468 | 6AL | 91.2-95.4 |  | 137.4-141.6 | - | - | - |
| *YrTtd-6AL.2* | IWB72189 | 6AL | 114.1-118.3 |  | 179.9-184.1 | - | - | - |
| *QYrTtd-6BS.1* | IWB23395 | 6BS | 5.9-10.1 |  | - | - | - | - |
| *QYrTtd-6BS.2* | IWB27151 | 6BS | 16.2-20.4 |  | 5.0-9.2 | - | - | - |
| *YrTtd-6BS* | IWB60487 | 6BS | 69.4-73.6 |  | 60.1-64.3 | IWB29373 | Liu et al. 2016 | IWB47211 |
| *YrTtd-6BL* | IWB12289 | 6BL | 74.4-78.6 |  | 81.5-85.7 | IWB55752 | Liu et al. 2016 | - |
| *YrTtd-7AS* | IWB61392 | 7AS | 6.6-10.8 |  | 8.2-12.4 | - | - | - |
| *YrTtd-7AL.1* | IWB21459 | 7AL | 117.6-121.8 |  | 99.2-103.4 | - | - | - |
| *YrTtd-7AL.2* | IWA1944 | 7AL | 132.2-136.4 |  | 100.4-104.6 | IWA808 | Liu et al. 2016 | - |
| *QYrTtd-7AL.1* | IWB25121 | 7AL | 190.9-195.1 |  | 166.1-170.3 | - | - | - |
| *QYrTtd-7AL.2* | IWA501 | 7AL | 198.7-202.9 |  | 182.9-187.1 | *QYr.cim-7AL_Avocet* | Rosewarne et al. 2012 | - |
| *YrTtd-7BS.1* | IWB71730 | 7BS | 28.1-32.3 |  | 21.5-25.7 | - | - | - |
| *YrTtd-7BS.2* | IWB13775 | 7BS | 60.6-64.8 |  | 41.7-45.9 | - | - | IWA3129, IWB41451, IWB70254, IWB69577 |
| *YrTtd-7BL* | IWB69807 | 7BL | 91.9-96.1 |  | 57.6-61.8 | IWB63652 | Liu et al. 2016 | - |
| *QYrTtd-7BL* | IWB72249 | 7BL | 191.1-195.3 |  | 155.3-159.5 | *Yr59* | Zhou et al. 2014 | - |

^a^ Chromosomal positions of identified QTL were based on the tetraploid wheat consensus map (Maccaferri et al. 2015a).

^b^ The identified QTL were mapped to the same chromosome arm based on the hexaploid integrated map (Maccaferri et al. 2015b) and the tetraploid wheat consensus map (Maccaferri et al. 2015a). The QTL characterized in our study were aligned to documented stripe rust resistance QTL by using the map positions on the hexaploid integrated map (Maccaferri et al. 2015b). References were listed for the documented stripe rust that overlap with QTL in our study.

^c^ The significant SNPs that commonly reside in the integrated map and consensus map based-confidence intervals.
